# First-Principles Investigation into Structural and Optoelectronic Properties of Mixed Halide Perovskites CsPbBr_3–*y*_X_*y*_ (X = I, Cl, F; y = 0, 1, 2, 3) Using GGA, mBJ, mBJ + SOC, and HSE Functionals

**DOI:** 10.1021/acsomega.6c06754

**Published:** 2026-09-08

**Authors:** Paraman Mahalaxmi, Vasu Veerapandy, Ponniah Vajeeston

**Affiliations:** † Department of Computational Physics, School of Physics, 29944 Madurai Kamaraj University, Madurai 625021, India; ‡ Department of Chemistry, Center for Materials Science and Nanotechnology, University of Oslo, Oslo 0371, Norway

## Abstract

This study presents a comprehensive density functional theory investigation into the structural, electronic, and optical properties of inorganic halide perovskites with the general formula CsPbBr_3–*y*_X_*y*_ (where X = I, Cl, F; and y = 0, 1, 2, 3). Using the Vienna Ab initio Simulation Package, electronic band structures were calculated employing a hierarchy of functionals: the generalized gradient approximation, Tran-Blaha-modified Becke-Johnson (TB-mBJ), TB-mBJ combined with spin–orbit coupling, and the hybrid HSE06 functional. Structural analysis confirms that substituting bromine with smaller halides such as fluorine (F) and chlorine (Cl) contracts the lattice, whereas iodine (I) substitution leads to lattice expansion, a trend consistent with the relative ionic radii. The results reveal systematic trends in bandgap tuning; incorporation of F and Cl widens the band gap, whereas I substitution narrows it. These changes are attributed to shifts in orbital interactions and varying electronegativities. Optical calculations demonstrate that F and Cl substitution decreases the low-energy electronic polarizability, yielding lower static real dielectric constants, indicating reduced electronic polarizability favorable for wide bandgap and UV-reflective coating components, whereas increasing I concentration raises the refractive index and stretches optical absorption deep into the visible range, matching solar illumination requirements. Spectroscopic limited maximum efficiency analysis of ten distinct compositions identifies CsPbI_3_ as the premier candidate, yielding a maximum theoretical spectroscopic efficiency of approximately 31% under radiative limits. These findings underscore halide substitution as an effective strategy for engineering the optoelectronic behavior of CsPbBr__3__-based perovskites for next-generation energy applications.

## Graphical abstract

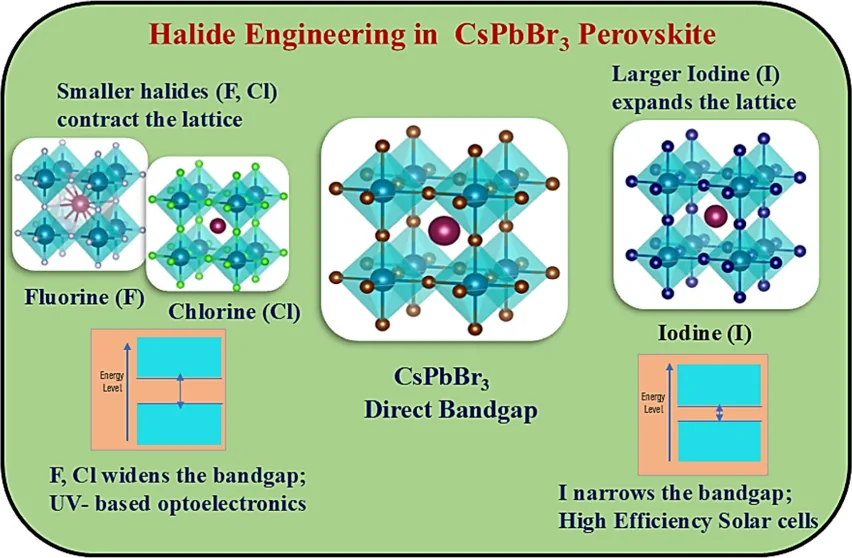

## 1 Introduction

Inorganic lead halide perovskites CsPbX_3_ (where X can be F, Cl, Br, or I) have emerged as promising materials for next-generation optoelectronics, due to their adjustable direct bandgaps, high absorption coefficients, and excellent charge transport properties.^1–6^ The ability to tune their electronic and optical characteristics through halide mixing enables precise bandgap engineering, facilitating device optimization across a broad spectral range.^7,8^ This capacity for halide substitution has positioned mixed halide perovskites as a revolutionary category of materials in high-performance solar cells, LEDs, and photodetectors.^9–11^ Recently Selmani et al. investigated the potential of these mixed systems. Investigations into CsSnBr_3‑x_Cl_*x*_ frameworks indicate that while CsSnBr_3_ and CsSnCl_3_ crystallize in cubic phases, mixed halides adopt tetragonal structures, with negative formation energies with GGA-PBE and HSE functionals.^12^ Furthermore, an increase in chlorine concentration has been shown to widen the bandgap and blue-shift optical absorption edges, enhancing potential for solar energy capture. Parallel studies by Kumari et al. on CsPbX_3_ (X = Cl, Br, I) intersubstituted variants have shown that larger halide ionic radii lead to a systematic bandgap tuning from approximately 1.64 to 3.04 eV.^13^ While compositions like CsPbCl_2_I offer a compelling balance of optical and mechanical properties, they also highlight that certain intersubstituted variants can exhibit mechanical instability.

Jen et al. present theoretical studies on the CsPbBr_*x*_I_3‑x_ series demonstrating that equilibrium lattice constants increase monotonically with iodine concentration due to the larger atomic radius of iodine.^14^ It has been noted that in these iodine-rich systems the bandgaps decrease significantly under strong SOC effects. This suggests that chemical composition and atomic displacements can collectively induce topological invariant phases in these perovskites. Ghaithan et al. examine the impact of chlorine substitution in CsPbBr_1–*x*_Cl_*x*_ perovskites through DFT calculations using the PBE-GGA and mBJ methods.^15^ Analysis reveals that increasing Cl concentration linearly decreases the unit cell volume and causes a widening of the band gap, ranging from 1.53 to 1.93 eV with PBE-GGA and 2.23 to 2.90 eV with mBJ, with high Cl content leading to a blueshift in optical transition. Bonding analyses in this system indicate that ionic character dominates in Cs-halide bonds, whereas Pb-halide bonds possess stronger covalent contributions, directly influencing material stability and electronic behavior.

While prior computational studies have examined Cl and I substitution in CsPbBr_3_, this work provides a unified, comprehensive framework that extends beyond existing literature through three distinct contributions: first, a systematic investigation of the under-explored F-substituted series (CsPbBr_3‑y_X_*y*_ where X = I, Cl, F) is presented, capturing structural and electronic variations that have received limited attention due to experimental synthesis challenges. Second, a rigorous comparative analysis across four distinct exchange-correlation frameworks (GGA, mBJ, mBJ + SOC, and HSE06) is deployed for the entire compositional series, enabling a direct assessment of functional reliability, spin–orbit coupling effects, and bandgap trends under identical computational constraints. Third, a first-principles spectroscopic limited maximum efficiency analysis is implemented to evaluate the theoretical photovoltaic potential as a function of absorber layer thickness, providing quantitative efficiency predictions that go well-beyond conventional bandgap assessments alone.

This study presents a systematic DFT investigation of ten distinct inorganic halide compositions, including CsPbBr_3_, CsPbBr_2_Cl, CsPbBrCl_2_, CsPbCl_3_, CsPbBr_2_F, CsPbBrF_2_, CsPbF_3_, CsPbBr_2_I, CsPbBrI_2_, and CsPbI_3_. A hierarchy of functionals is employed within the VASP framework, utilizing the GGA, TB-mBJ, and HSE06 functionals. To capture the essential relativistic effects of the heavy Pb cations without the prohibitive computational overhead of hybrid-SOC combinations, we integrate SOC specifically with the mBJ functional (mBJ+SOC). By providing a comprehensive analysis of structural, electronic, and optical responses, including SLME data, this work provides a path for the design and optimization of mixed halide perovskites for next-generation solar cells.

## 2 Computational Method

Density functional theory calculations were conducted using the projector-augmented wave (PAW) method, as implemented in the Vienna Ab Initio Simulation Package (VASP).^16,17^ The following valence electron configurations were treated as valence states for the pseudopotentials: Cs (5s^2^ 5p^6^ 6s^1^), Pb (6s^2^ 5d^10^ 6p^2^), F (2s^2^ 2p^5^), Cl (3s^2^ 3p^5^), Br (4s^2^ 4p^5^), and I (5s^2^ 5p^5^). Structural optimizations, including full relaxation of unit cell volumes and atomic positions, were performed using the Perdew–Burke–Ernzerhof (PBE) parametrized GGA.^18^ The unit cell volume and atomic positions were fully relaxed until the residual forces on each atom were below 0.01 eV/Å, with a total energy convergence criterion of 10^–6^ eV. A plane wave energy cutoff of 520 eV was employed. A 6 × 6 × 6 k-point mesh was used for structural optimization. For the calculation of the DOS (Density of States), the Brillouin zone was sampled with a denser 8 × 8 × 8 k-point mesh. The plane-wave cutoff energy convergence and the k-point convergence for systems CsPbBr_3_, CsPbBr_2_F, CsPbCl_3_, and CsPbBrI_2_ are calculated and presented in Figure S5 of Supporting Information. Electronic properties were evaluated using PBE–GGA and TB-mBJ potentials.^19^ To capture the relativistic effects of heavy Pb atom, SOC was included in the mBJ formalism (mBJ+SOC). The inclusion of SOC is important as it induces relativistic splitting of the Pb conduction band states, thereby shifting the conduction band downward and significantly narrowing the bandgap.^20^ For a more refined description of the electronic structure, the Heyd, Scuseria, and Ernzerhof (HSE06) hybrid functional was employed, utilizing a standard screening parameter of 0.2 Å^–1^ and 25% exact Hartree–Fock exchange.^21,22^ We explicitly note that this parameter set represents the canonical, nonempirical definition of the standard HSE06 functional and natively defaulted within the VASP framework.^23^ HSE06 applies a screened short-range exchange mechanism that effectively mitigates self-interaction errors while significantly reducing computational overhead, thereby providing an optimal balance of numerical precision and efficiency for calculating complex perovskite frameworks.^24^ The DOS and optical properties were calculated using TB-mBJ potential framework. The optical properties were examined by calculating the complex dielectric constant, utilizing a 10 × 10 × 10 k-point mesh for Brillouin zone sampling. To calculate the SLME, the optical absorption coefficients (α) were evaluated as a function of absorber layer thickness using the VASPKIT tool. The input parameter files containing these absorption coefficients were extracted from the TB-mBJ potential framework (without SOC).

## 3 Results and Discussion

### 3.1 Structural Parameters

The crystal structures of the mixed halide inorganic perovskite are illustrated in Figure 1. The CsPbX_3_ (X = F, Cl, Br, I) cubic perovskite structure is characterized by a three-dimensional network of regular corner-linked PbX_6_ octahedra. Within this symmetric lattice, the Pb cation is centrally positioned inside each octahedron and the Cs cation occupies a larger cuboctahedra cavity situated between these octahedral units. These octahedra share corners across all three spatial dimensions, forming a robust and highly interconnected framework.^25,26^ The crystalline structures of CsPbF_3_, CsPbBr_3_, CsPbCl_3_, and CsPbI_3_ are modeled in a high-symmetry cubic phase with the space group *Pm* 3̅ *m* (No. 221). In this configuration, Cs atoms occupy the 1*b* Wyckoff position at (1/2, 1/2, 1/2), Pb atoms are located at 1*a* (0, 0, 0), and the halogen atoms reside at 3*d* positions at (1/2, 0, 0). For the mixed-halide systems, the substitution of halides leads to a symmetry reduction from cubic to a tetragonal phase with space group *P*4/*mmm* (No. 123). In the tetragonal structure, Cs and Pb atoms located at the 1*d* (1/2, 1/2, 1/2) and 1*a* (0, 0, 0) positions, respectively. The halogen atoms distribute such that two halide atoms occupy the 2*f* (0, 1/2, 0) site and a single halide atom occupies the 1*b* (0, 0, 1/2) site. These calculated cubic and tetragonal structures serve as idealized reference models rather than true ambient equilibrium phases. These calculated configurations show how changing the halide composition directly affects lattice relaxation, and orbital hybridization, without being complicated by extra structural changes. The octahedral and tolerance factor for these perovskites are calculated and presented in Table S1 of Supporting Information. The lattice constants and bulk moduli, determined by fitting energy-volume data to the Birch–Murnaghan fourth-order equation of state and the energy-volume (E-V) curve, are presented in Figures S1–S4 of Supporting Information.^27,28^ The structural parameters are summarized in Table 1.

**Figure 1:**
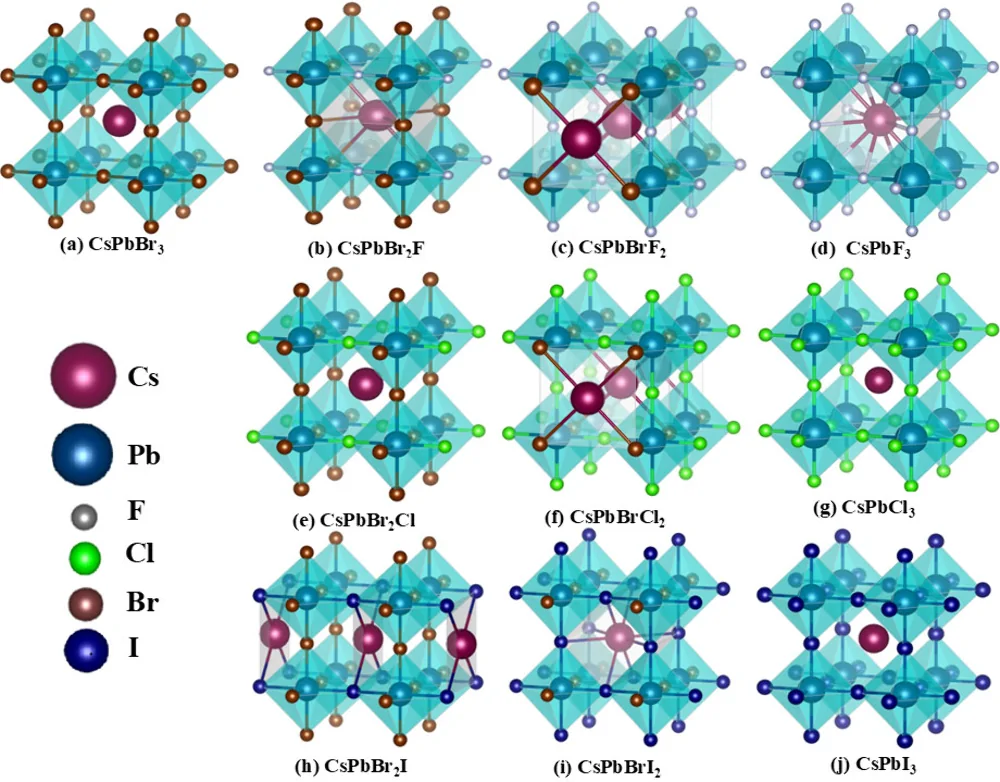
Crystal structure of mixed halide inorganic perovskite.

**Table 1:** Theoretical Lattice Parameters (Å), Unit-Cell Volume V (Å^3^), Bulk Modulus B (GPa), and Formation Energy (eV) for CsPbBr_3–*y*_X_*y*_ ^a^ ^,^ ^a^

**S. No.**	**System**	**Lattice constant (Å)**	**Volume (Å** ^**3**^ **)**	**Bulk modulus (GPa)**	**Formation Energy (eV)**
1.	CsPbBr_3_ *(Pm* 3̅ *m)*	*a* = 6.008	214.1	19.2	–13.46
		*a* = 5.874 ^15^	214.9 ^30^	17.2 ^30^	–16.07 ^30^
		*a* = 5.990 ^30^	214.7 ^33^		
		*a* = 5.988 ^33^			
2	CsPbBr_2_F *(P4/mmm)*	*a* = 6.134	180.8	22.8	–15.51
		*c* = 4.794			
3	CsPbBrF_2_ *(P4/mmm)*	*a* = 4.960	152.1	26.8	–17.35
		*c* = 6.150			
4	CsPbF_3_ *(Pm* 3̅ *m)*	*a* = 4.904	117.5	39.1	–19.65
		*a* = 4.908 ^34^	118.1 ^34^	38.8 ^34^	
		*a* = 4.973 ^35^		36.2 ^35^	
		[*a* = 4.25][Bibr ref36]			
5	CsPbBr_2_Cl *(P4/mmm)*	*a* = 6.021	207.4	19.5	–13.98
		*a* = 5.784 ^15^		26.0 ^13^	
		*c* = 5.715			
		*c* = 5.748 ^15^			
6	CsPbBrCl_2_ *(P4/mmm)*	*a* = 5.730	198.2	20.5	–14.51
		*a* = 5.965 ^15^		33.0 ^13^	
		*c* = 6.028			
		*c* = 5.695 ^15^			
7	CsPbCl_3_ *(Pm* 3̅ *m)*	*a* = 5.734	187.0	21.8	–15.05
		*a* = 5.605 ^15^	187.9 ^33^		
		*a* = 5.728 ^33^			
		[*a* = 5.60][Bibr ref37]			
8	CsPbBr_2_I *(P4/mmm)*	*a* = 6.005	231.8	17.1	–12.87
		*a* = 6.132 ^30^	230.6 ^30^	14.77 ^30^	–15.35 ^30^
		*c* = 6.419			
9	CsPbBrI_2_ *(P4/mmm)*	*a* = 6.414	246.6	15.8	–12.31
		*a* = 6.263 ^30^	245.6 ^30^	13.86 ^30^	–14.75 ^30^
		*c* = 5.982			
10	CsPbI_3_ *(Pm* 3̅ *m)*	*a* = 6.400	261.4	14.5	–11.76
		*a* = 6.382 ^30^	259.9 ^30^	12.49 ^30^	–14.23 ^30^

a [bolded] represent reported experimental values.

b X = I, Cl, F; y = 0, 1, 2, 3.

Analysis of the computed lattice constants indicates that substituting Br with smaller halides (F and Cl) leads to systematic contraction, whereas substitution with larger I anion leads to an expansion of the lattice.^14^ This trend follows the principle that smaller ionic radii reduce the unit cell volume, whereas larger radii yield an expansion. The calculated lattice constants match well with previously reported literature values.^13–15,29,30^ The bulk moduli (B) reported in Table 1 were calculated by fitting calculated total energy-volume (E-V) data to the fourth-order Birch–Murnaghan equation of state. This E-V fitting approach is highly reliable for tracking isotropic compression volume trends. The calculated values follow the well-established mechanical trend where smaller, less polarizable halide ions (F) yield significantly stiffer lattices (higher *B*) compared to the softer, more compliant iodine variants. The calculated B value of 14.5 GPa for CsPbI_3_ matches the experimental value of 14 ± 3 GPa derived from high-pressure synchrotron X-ray diffraction measurements.^31^ For CsPbF_3_, explicit experimental mechanical moduli are currently missing from the literature due to the practical difficulties of growing macro-crystals stable enough for high-pressure testing. Our computed value of 39.1 GPa strictly follows the expected physical trend where lattice compressibility is inversely proportional to the ionic radius of the constituent anions. Replacing the bulky, highly polarizable I ion with the smaller, highly electronegative F ion shrinks the lattice framework and yields significantly stiffer Pb–F bonds, naturally driving the observed increase in mechanical resistance against uniform compression. The bulk modulus decreases as the lattice constant increases; this is attributed to the increased bond distances associated with larger lattices, which results in relatively weaker bonding.^13^ The electronic compressibility of the atoms, which is proportional to the fourth power of the atomic radius, may contribute significantly to this observed reduction in bulk modulus.^32^

In the pristine system (CsPbBr_3_), the substitution of halides breaks the cubic isotropic symmetry, driving a phase transition to the tetragonal *P4/mmm* space group. Because two halide atoms occupy the 2*f* sites and one occupies the 1*b* site, the chemical environment becomes highly anisotropic. The calculated *c/a* < 1 ratio signifies a distinct out-of-plane lattice compression along the *c*-axis balanced by an in-plane structural relaxation along the *a*-axis.^38^ From the structural parameters summarized in Table 1, the mixed halide systems demonstrate unique directional relaxations. Upon moving from the highly symmetric cubic Pm3̅m phase to the mixed tetragonal *P4/mmm* phase, structural anisotropy is introduced due to the uneven ordering of the halogens across the 2*f* and 1*b* Wyckoff positions. This structural symmetry reduction leads to a *c/a* < 1 ratio in compositions like CsPbBr_2_F and CsPbBr_2_Cl. While the in-plane lattice parameter *a* expands slightly compared to pristine CsPbBr_3_, the out-of-plane *c*-axis undergoes a pronounced compression.^39,40^ The overall unit cell volume contracts monotonically as smaller halides (F, Cl) substitute bromine (214.4 Å^3^ → CsPbBr_3_, 207.4 Å^3^ → CsPbBr_2_Cl and 180.8 Å^3^ → CsPbBr_2_F), confirming that the absolute structural density follows the expected ionic radii trends. The formation energy (Δ*E*_*f*_) in eV per formula unit of these systems was calculated using the following expression:

$$\Delta E_{f}=E_{\mathrm{total}}\left({\mathrm{CsPbX}}_{3}\right)-\left[E_{\mathrm{total}}\left(\mathrm{Cs}\right)+E_{\mathrm{total}}\left(\mathrm{Pb}\right)+3\times E_{\mathrm{total}}\left(X\right)\right]$$

where *E*_total_ represents the total energy of the optimized CsPbX_3_ unit cell and *E*_total_ (Cs), *E*_total_ (Pb), and *E*_total_ (X) are the total energies per atom of the pure elemental reference phases.^30^ The pure elemental reference phases were chosen according to their most stable, structurally relaxed ground states at 0 K within the VASP projector-augmented wave (PAW) pseudopotential framework. The reference structures were modeled as body-centered cubic (BCC) for Cs, face-centered cubic (FCC) for Pb, and high-symmetry molecular crystals optimized at 0 K for the halogens, ensuring an accurate and consistent baseline for isolated atomic environments during the evaluation of cohesive formation energies. The formation energies for all investigated halides investigated are presented in Table 1, where it is noted that the formation energies are negative for all pristine and mixed halides. The shift toward less negative formation energies from CsPbBr_3_ (−13.46 eV) to CsPbI_3_ (−11.76 eV) indicates a systematic reduction in the chemical binding and cohesive energy relative to the pure elemental reference phases. This variation implies that iodine-rich compositions are energetically less favorable to form compared to their bromide-rich counterparts. This behavior is physically consistent with the increased lattice strain introduced when forcing the significantly larger I ionic radius into the corner-linked PbX_6_ octahedral framework, which slightly destabilizes the local binding network.^30^

### 3.2 Electronic Parameters

The electronic band structures of CsPbBr_3‑y_X_*y*_ (where X = I, Cl, F; y = 0, 1, 2, 3) were investigated using a systematic hierarchy of DFT calculations. The PBE–GGA functional, while computationally efficient, tends to underestimate bandgaps due to its semilocal nature, which limits its predictive reliability for lead-based perovskites.^41^ To address this, TB-mBJ potential was utilized, as it yields bandgap estimates that show good consistency with experimental observations.^20^ Figure 2 shows the bandgap structure of CsPbBr_3_ where GGA produced a bandgap of 1.82 eV, TB-mBJ potential produced a bandgap of 2.12 eV, and HSE06 functional yields a bandgap of 2.83 eV, which noticeably exceeds the experimental benchmark of ∼ 2.3 eV. This overestimation is a well-known behavior in heavy Pb-based systems. Hybrid functionals like HSE06 inject a fixed amount of exact Hartree–Fock exchange math to artificially widen standard electronic gaps. However, heavy-metal perovskites possess strong, highly mobile electron clouds that natively screen (or shield) these electrical interactions. Because standard HSE06 does not fully account for this internal material shielding, it overcorrects the states, pushing the conduction band too far upward and resulting in values that are higher than the true experimental baseline.^21^

**Figure 2:**
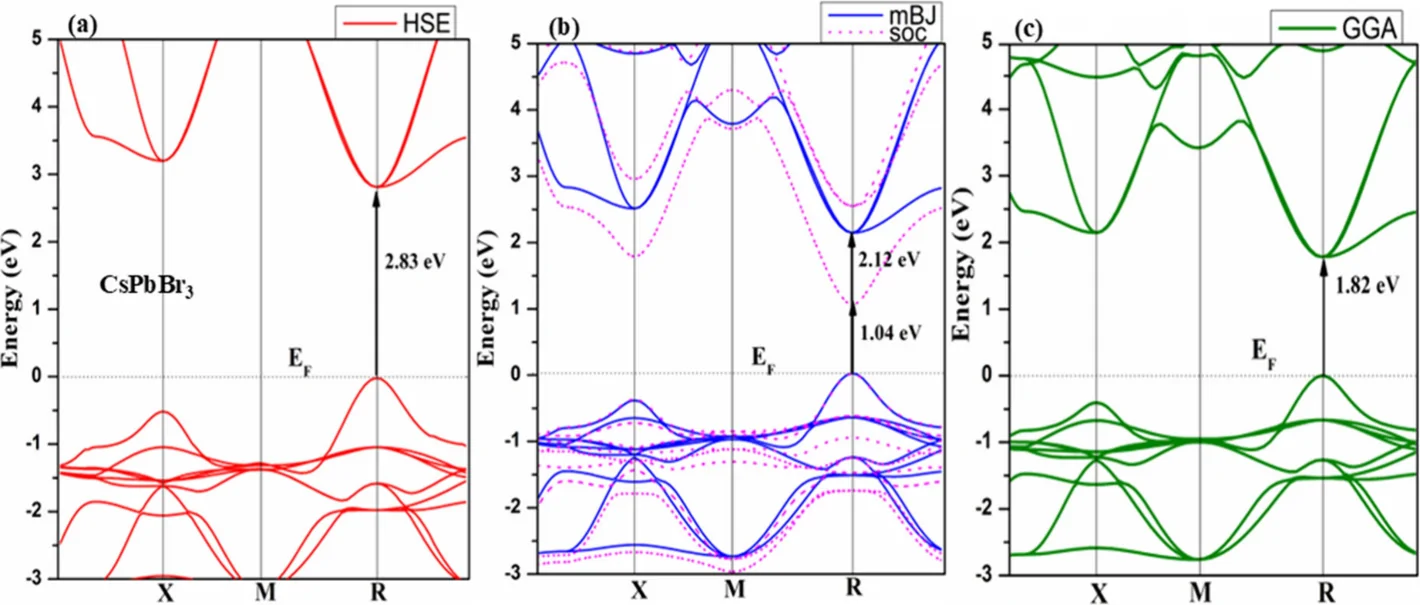
Band structure of CsPbBr_3_ calculated using (a) HSE06 and (b) mBJ potential without/with SOC and (c) GGA.

The significant influence of relativistic effects is visible when evaluating the band structures calculated with the inclusion of spin–orbit coupling (mBJ+SOC).^15,30^ As shown in Figure 2(b), the inclusion of SOC induces a severe relativistic splitting of the Pb 6*p*-derived conduction band states. This splitting causes a major downward shift of the conduction band minimum (CBM), effectively reducing the calculated electronic bandgap of CsPbBr_3_ to1.04 eV.^42,43^ The CBM splits into a lower-energy 2-fold-degenerate state (*p*_1/2_, associated with light electrons) and a higher-energy 4-fold-degenerate state (*p*_3/2_, associated with heavy electrons).^44,45^ This large shift represents a severe underestimation compared to experimental measurements (∼ 2.3 eV). This behavior is a consequence of a broken error cancellation: standard TB-mBJ closely approximates the experimental gap (2.12 eV) by completely omitting the relativistic CBM downshift. When SOC is explicitly applied without the long-range self-interaction corrections found in advanced hybrid functionals, it leads to an overcorrection of the conduction band edge. The calculated bandgap values are compared with reported values and listed in Table 2. The band structures for the mixed halides are shown in Figure 3, Figure 4, and Figure 5. For consistency and easier comparison, the Fermi level was set to zero in all cases. Across all CsPbBr_3–*y*_X_*y*_ compositions, both the valence band maximum (VBM) and conduction band minimum (CBM) are located at the high-symmetry R point, confirming a consistent direct bandgap nature. The effective masses of electrons (*m*_e_^*^) at the CBM and holes (*m*_h_^*^) at the VBM were evaluated along the R → Γ for all ten systems and presented in Table S2 of Supporting Information.

**Figure 3:**
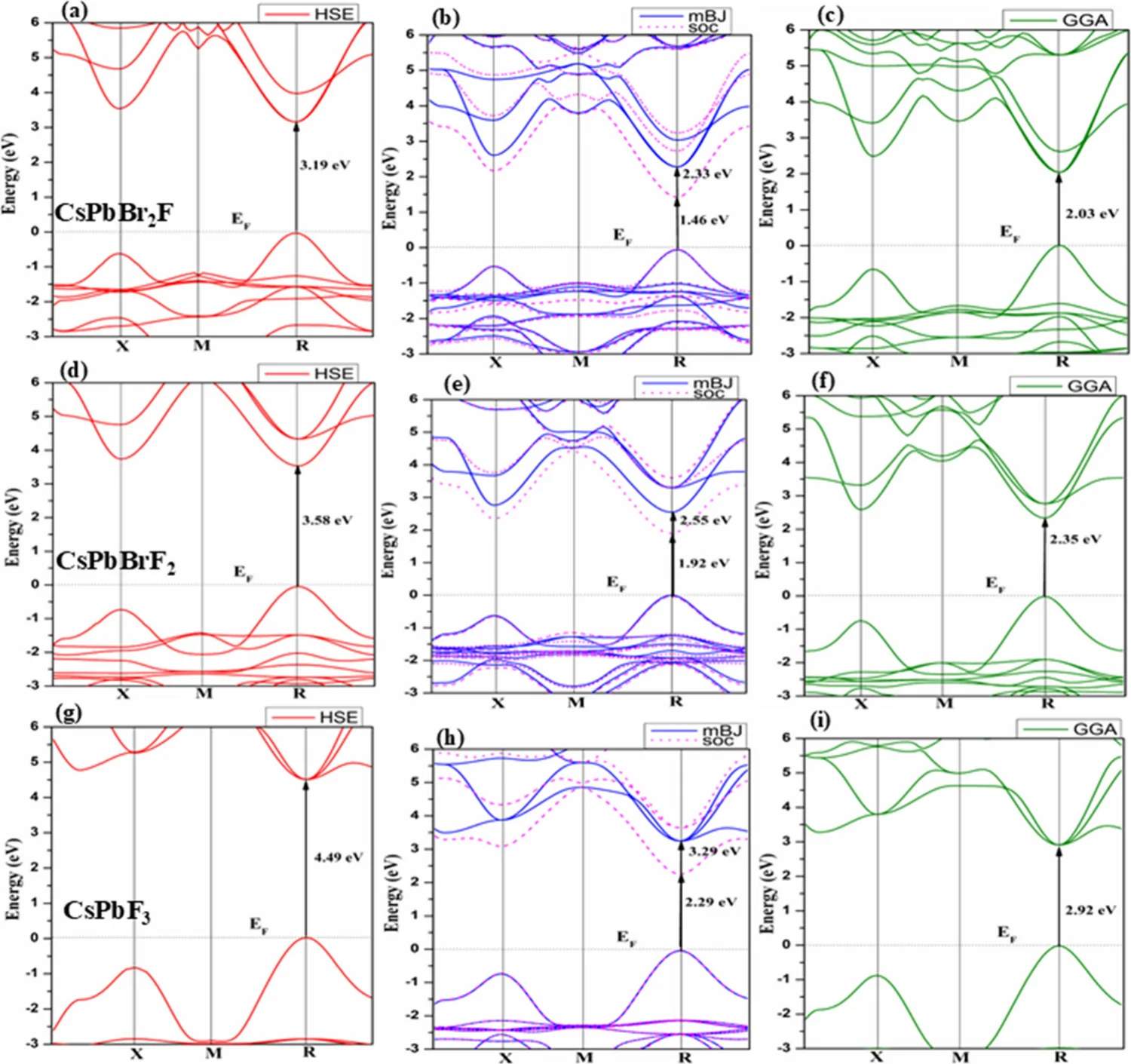
Band structure of (a-c) CsPbBr_2_F, (d-f) CsPbBrF_2_, and (g-i) CsPbF_3_ calculated using HSE06, mBJ potential without/with SOC and GGA.

**Figure 4:**
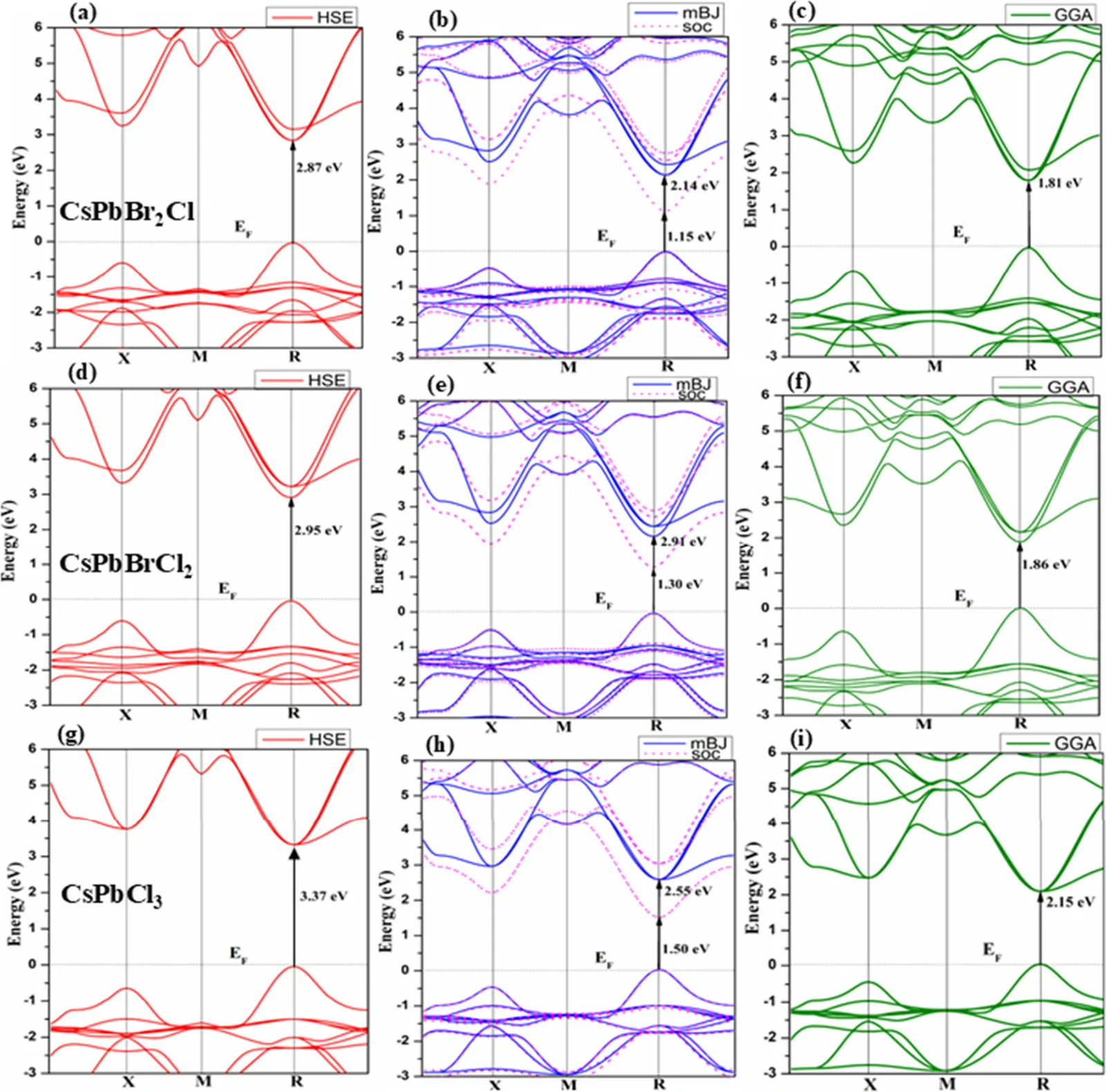
Band structure of (a-c) CsPbBr_2_Cl, (d-f) CsPbBrCl_2_, and (g-i) CsPbCl_3_ calculated using HSE06, mBJ potential without/with SOC and GGA.

**Figure 5:**
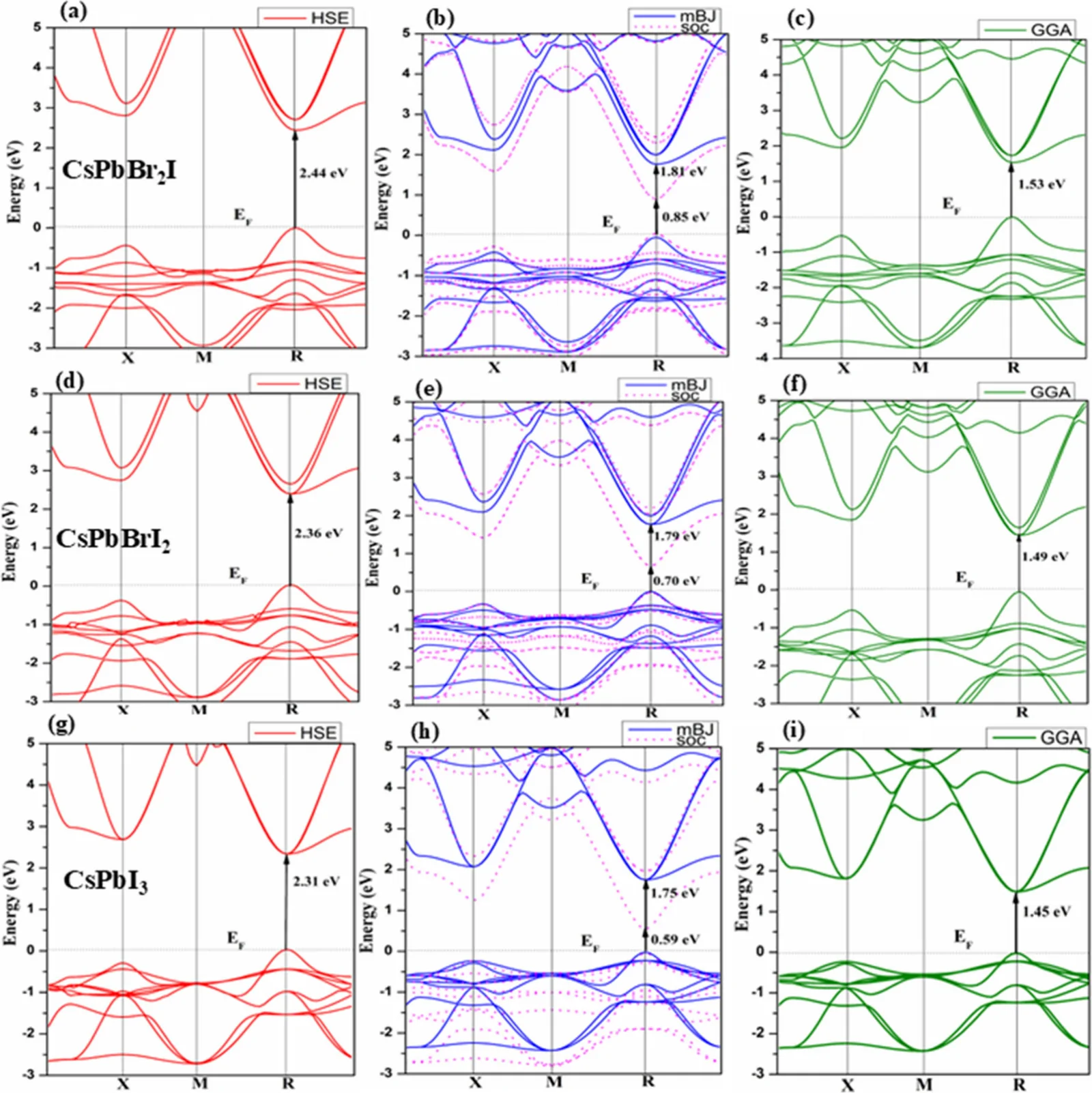
Band structure of (a-c) CsPbBr_2_I, (d-f) CsPbBrI_2_, and (g-i) CsPbI_3_ calculated using HSE06, mBJ potential without/with SOC and GGA.

**Table 2:** Bandgap *E*_*g*_ (eV) Values for CsPbBr_3–*y*_X_*y*_ ^a^ ^,^ ^a^

**S. No.**	**System**	**HSE06 (eV)**	**mBJ (eV)**	**mBJ + SOC (eV)**	**GGA (eV)**
1	CsPbBr_3_	2.83	2.12	1.04	1.82
		2.51 ^33^	2.29 ^13^	1.05 ^15^	1.76 ^14^
			2.23 ^15^		1.53 ^15^
			2.36 ^48^		1.67 ^33^
					1.66 ^30^
2	CsPbBr_2_F	3.19	2.33	1.46	2.03
3	CsPbBrF_2_	3.58	2.55	1.92	2.35
4	CsPbF_3_	4.49	3.29	2.29	2.92
		3.71 ^49^	[**3.68**][Bibr ref36]		2.99 ^47^
					2.98 ^35^
5	CsPbBr_2_Cl	2.87	2.14	1.15	1.81
		2.72 ^33^	2.55 ^13^	1.20 ^15^	1.56 ^15^
			2.40 ^15^		1.78 ^33^
6	CsPbBrCl_2_	2.95	2.19	1.3	1.86
		2.89 ^33^	2.74 ^13^	1.52 ^15^	1.71 ^15^
			2.59 ^15^		1.89 ^33^
7	CsPbCl_3_	3.37	2.55	1.5	2.15
		3.11 ^33^	3.09 ^13^	1.69 ^15^	1.93 ^15^
		[**3.00**][Bibr ref50]	2.90^15^		2.02^33^
			3.10 ^48^		2.17 ^48^
8	CsPbBr_2_I	2.44	1.81	0.85	1.53
			2.09 ^13^		1.52 ^14^
					1.48 ^30^
					
9	CsPbBrI_2_	2.36	1.79	0.7	1.49
			1.91 ^13^		1.36 ^30^
					1.40 ^14^
10	CsPbI_3_	2.31	1.75	0.59	1.45
		2.03 ^51^	1.64 ^13^		1.47 ^14^
			1.75 ^48^		1.31 ^30^
					1.47 ^48^

a X = I, Cl, F; y = 0, 1, 2, 3.

b [bolded] Values represent experimental values.

The band structures of CsPbBr_2_F, CsPbBrF_2_, and CsPbF_3_, calculated using HSE06, mBJ, and GGA functionals, are shown in Figure 3, revealing a clear trend as the F content increases, the bandgap values increase. For the F-substituted compounds, the bandgap increases from 2.33 eV (CsPbBr_2_F) to 3.29 eV (CsPbF_3_) with mBJ. The trend displays a slight nonlinearity due to localized lattice strain which is explained using a bowing parameter in section 5 of Supporting Information. Also, the rise in the bandgap with increasing F composition is due to the smaller ionic radius of fluorine compared to bromine, which leads to the formation of shorter and stronger Pb–F bonds within the crystal lattice. This enhancement in bond ionic character contracts the lattice and isolates electronic states, reducing the orbital overlap between Pb and the F atoms. The orbital hybridization between Pb 6*s*/6*p* and F 2*p* valence band weakens, which reduces the structural band dispersive interaction that reduces the bandgap.^46,47^

The replacement of bromine by chlorine in CsPbBr_3_ perovskites leads to a notable increase in the bandgap, as illustrated in Figure 4. The HSE-calculated bandgap for CsPbBr_2_Cl is 2.87 eV, whereas CsPbCl_3_ exhibits a wider bandgap of 3.37 eV. This trend is attributed to the fact that chlorine ions are more electronegative and possess a smaller ionic radius compared with bromine ions, resulting in a substantial broadening of the band gap. This phenomenon aligns with the trends reported in previous theoretical studies.^13,15,33^ The observed increase in the bandgap with decreasing halide ionic radius can be rationalized by considering the balance between atomic size and the electrostatic attraction between the nucleus and electrons.

The substitution of bromine by iodine in the CsPbBr_3_–CsPbI_3_ series results in a marginal narrowing of the bandgap, as illustrated in Figure 5, with values changing from 1.81 eV for CsPbBr_2_I to 1.75 eV for CsPbI_3_ under the mBJ framework. This minimal variation indicates that the electronic gap reaches a stabilized plateau upon high iodine incorporation. Hence, the bandgap modulation reflects the combined influences of ionic size, electronegativity, and relativistic effects, which are crucial for tailoring the electronic properties of halide perovskites. A systematic variation in bandgap values of mixed halide (CsPbBr_3–*y*_X_*y*_) perovskites calculated using GGA, mBJ, mBJ+SOC, and HSE06 functionals is illustrated in Figure 6.

**Figure 6:**
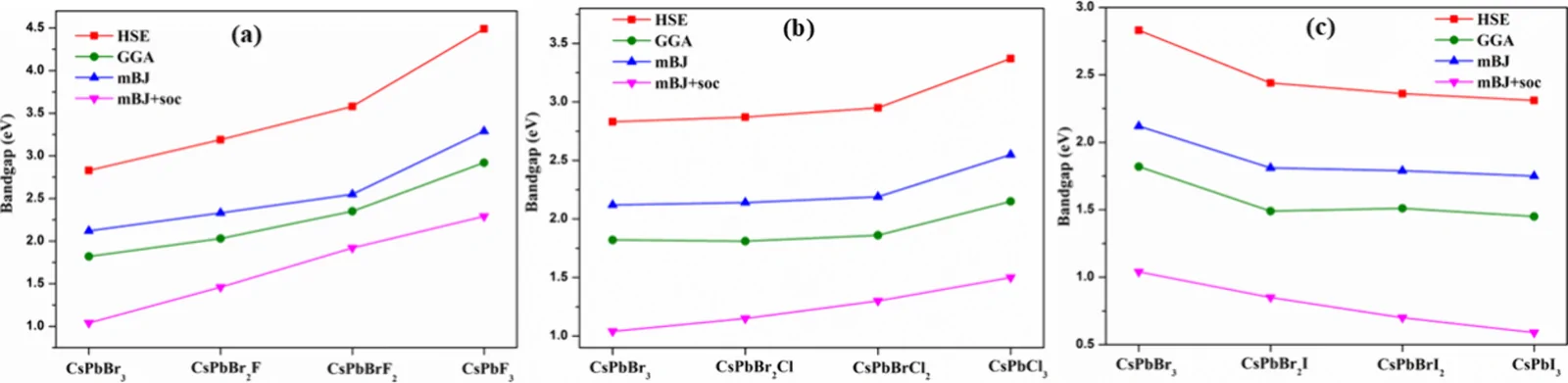
Variation in bandgap values of mixed halide perovskite calculated using the GGA, mBJ, mBJ+SOC, and HSE06 functionals

### 3.3 Density of States

To gain a comprehensive understanding of the electronic structures and bonding characteristics of CsPbBr_3‑y_X_*y*_ perovskites, an analysis of the total density of states (TDOS) and the partial density of states (PDOS) was conducted. The TDOS and PDOS for CsPbBr_3_ are illustrated in Figure 7, and for mixed halides they are illustrated in Figure 8, Figure 9, and Figure 10. The figure clearly shows a gap between the valence and conduction bands, reaffirming the semiconducting behavior of these materials. In all plots, the Fermi energy level (E_F_) is set to 0 eV as indicated by the vertical dashed line.

**Figure 7:**
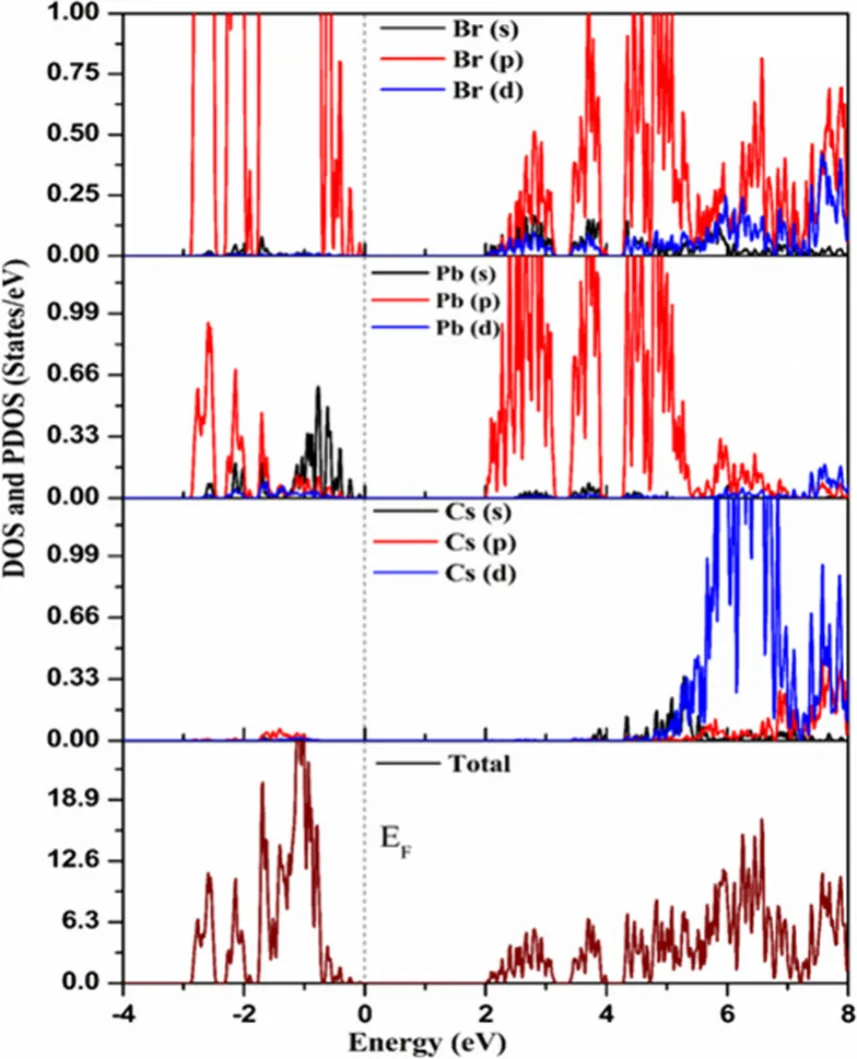
Calculated total density of states and projected density of states of CsPbBr_3_.

**Figure 8:**
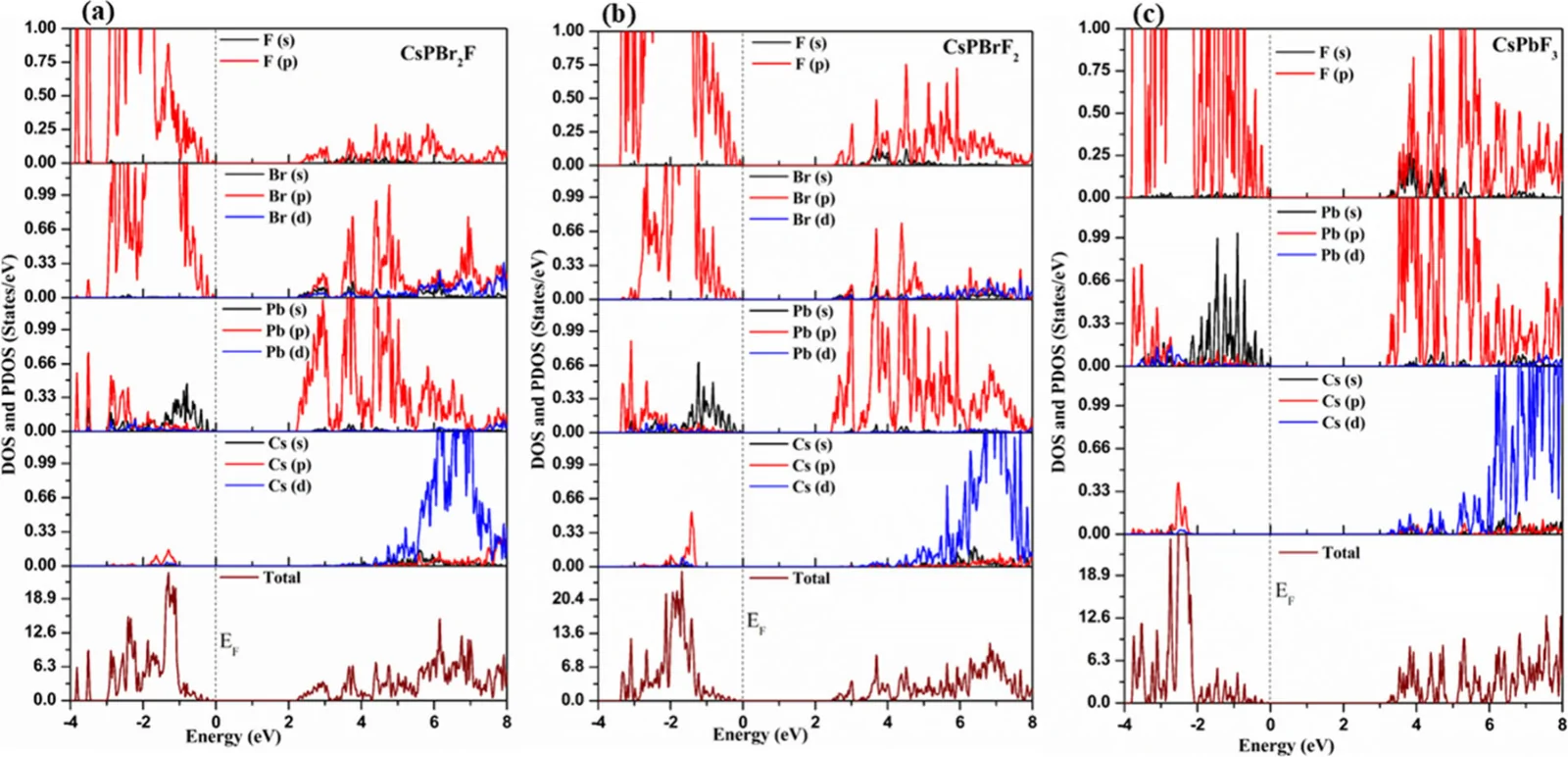
Calculated DOS and PDOS for (a) CsPbBr_2_F, (b) CsPbBrF_2_, and (c) CsPbF_3_.

**Figure 9:**
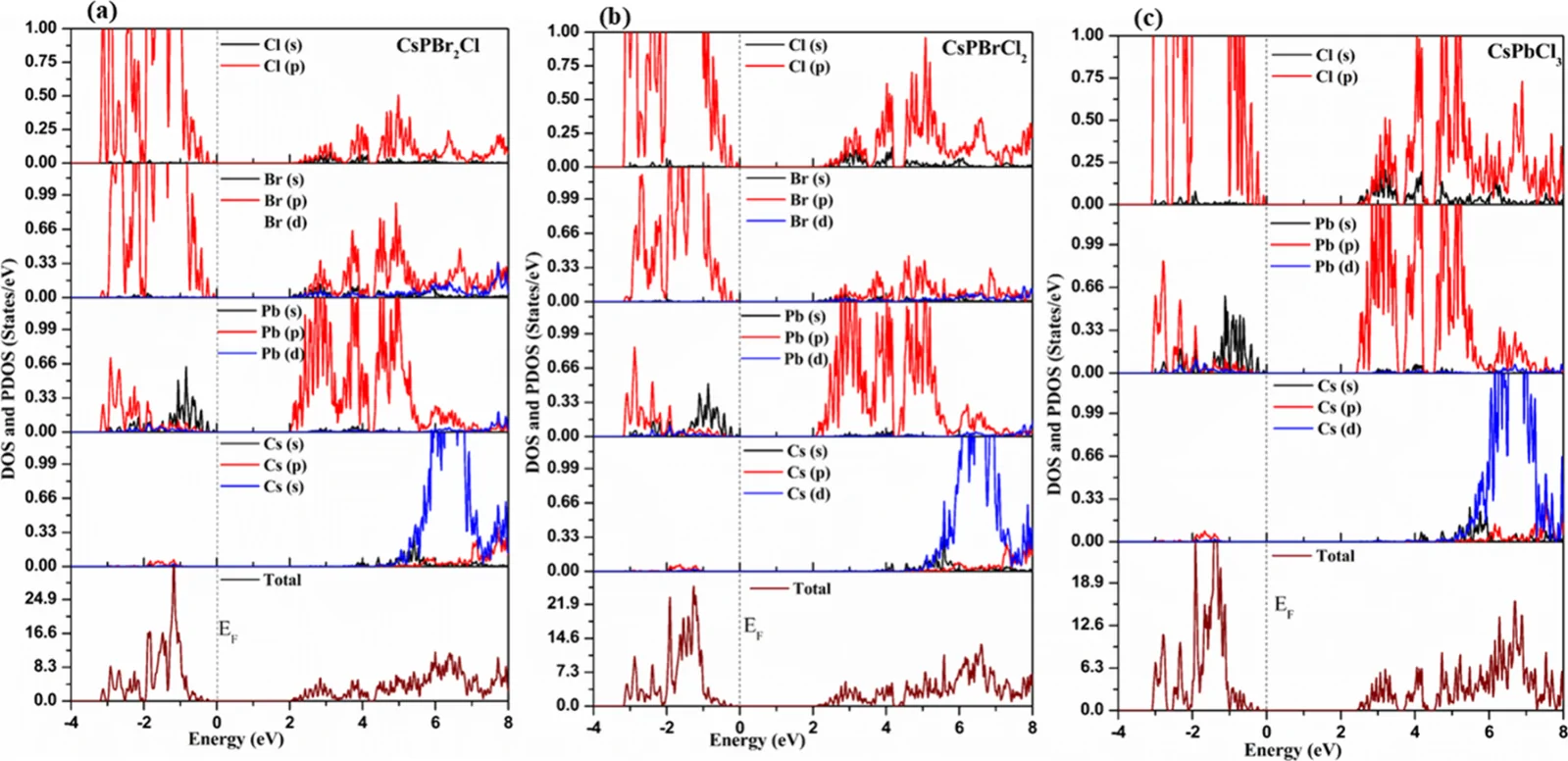
Calculated DOS and PDOS for (a) CsPbBr_2_Cl, (b) CsPbBrCl_2_, and (c) CsPbCl_3_.

**Figure 10:**
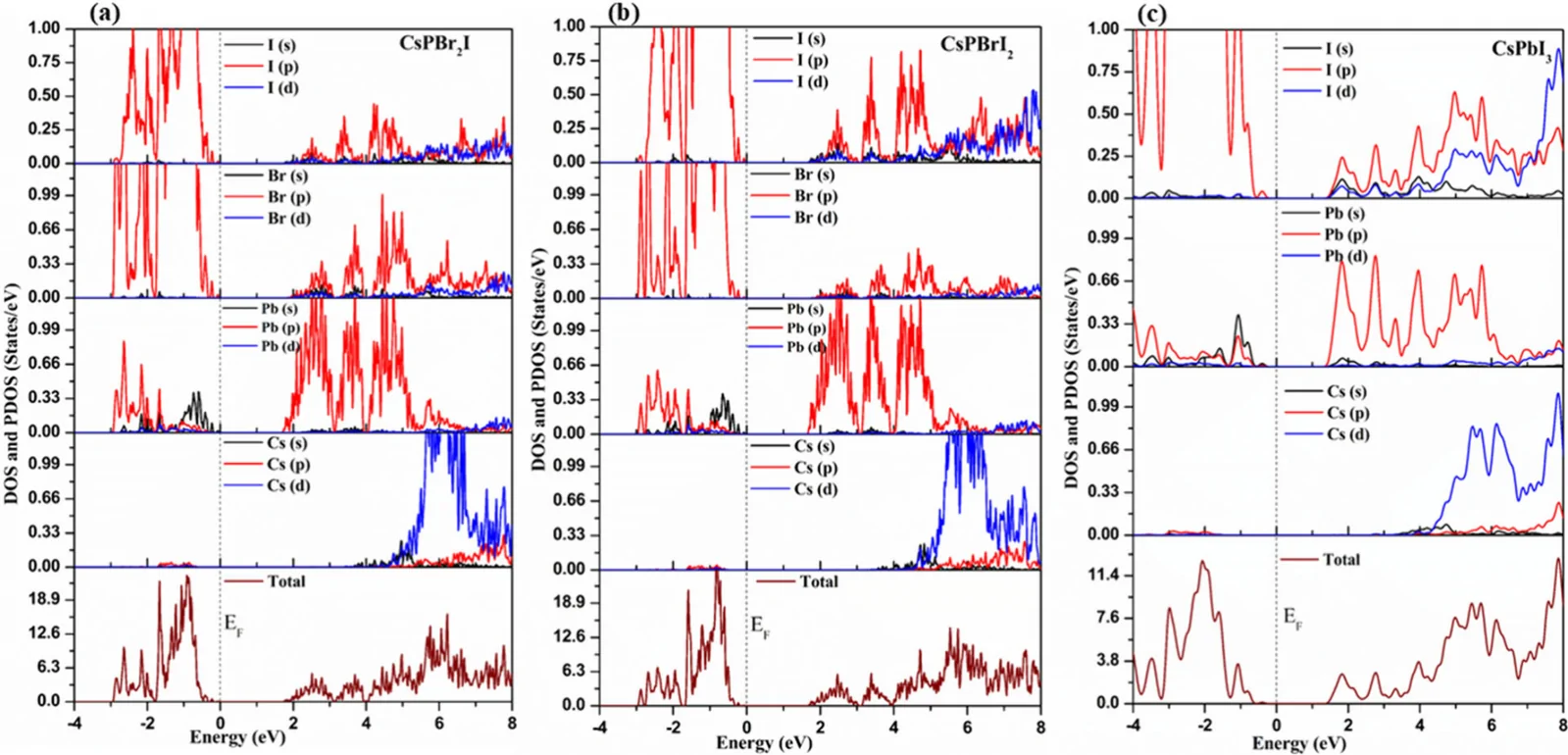
Calculated DOS and PDOS for (a) CsPbBr_2_I, (b) CsPbBrI_2_, and (c) CsPbI_3_.

The DOS for CsPbBr_3_ is illustrated in Figure 7. It reveals that the VBM is characterized by strong contributions between Pb *s* orbitals and Br *p* orbitals, with secondary contributions from Pb *p* states. The CBM is primarily composed of Cs *d* and Pb *p* orbitals, with minor contributions from Pb *d* and Br *s* orbitals.^52^

Figure 8 illustrates the total and partial densities of states for CsPbBr_2_F, CsPbBrF_2_, and CsPbF_3_, demonstrating the evolution of the electronic structure increasing fluorine content. In all three compounds, the valence band edge remains determined by the hybridization of Pb *s* states with halide *p* orbitals. As fluorine content increases from CsPbBr_2_F to CsPbF_3_, the contribution of Br *p* states is gradually replaced by F *p* states. In CsPbF_3_, the VBM is dominated by Pb *s* and F *p* states, with the F *p* manifold shifting to lower energy levels. This stabilization of the valence band, combined with a concurrent rise in the CBM, primarily derived from Cs *d* and Pb *p* orbitals. In the F-substituted series, the F 2*p* states appear approximately 2–3 eV below the Br 4*p* states due to fluorine’s higher electronegativity. This energy stabilization lowers the VBM when F substitutes for Br, because the VBM is composed primarily of halide *p* states hybridized with Pb 6*s*. Since the CBM remains relatively unchanged (dominated by Pb 6*p* states), lowering the VBM increases the energy separation between VBM and CBM, thereby widening the bandgap. Figure 9 illustrates the DOS profiles for CsPbBr_2_Cl, CsPbBrCl_2_, and CsPbCl_3_. A similar trend is observed in the Cl-substituted systems. The VBM originates from the hybridization of Pb *s* orbitals with Cl and Br *p* orbitals along with minor contributions from Pb *p* and *d* orbitals. Increased chlorine content leads to downshift in the VBM due to the higher electronegativity of Cl, resulting in a widened bandgap compared to the bromide-rich counterparts. CsPbCl_3_ exhibits the largest bandgap within this series. Figure 10 presents the DOS for CsPbBr_2_I and CsPbBrI_2_, CsPbI_3_. The DOS analysis reveals that valence bands are predominantly formed from Pb *s* orbitals hybridized with the halide *p* orbitals, with minor Pb *s* and *p* contributions near the Fermi level. The introduction of the larger iodine ion causes an upward shift in the valence band edge and enhanced spin–orbit coupling effects, which facilitate bandgap narrowing.^15^

The role of the Cs^+^ cation across all compositions can be clearly verified from the PDOS profiles in Figures 7 - [Fig fig10]. The Cs atomic states do not contribute to either the VBM or the CBM, confirming that its behavior is purely ionic within this lattice framework. Instead, the Cs *d* orbitals form a prominent electronic manifold deep within the upper conduction bands, appearing at energies greater than 5 eV above the Fermi level. This demonstrates that while the Cs^+^ ion does not directly influence the fundamental optoelectronic band edges, it plays a vital, passive role in structurally stabilizing the three-dimensional corner-sharing network of the PbX_6_ octahedra. The DOS characteristics demonstrate how the halide composition influences the electronic structure through orbital hybridization and relativistic interactions.

### 3.4 Optical Properties

The investigation of optical properties, including the complex dielectric function ε­(ω), absorption coefficient α­(ω), refractive index *n*(ω), extinction coefficient *k*(ω), and reflectivity R­(ω), is essential for characterizing the light–matter interactions within CsPbBr_3‑y_X_*y*_ perovskites. The complex dielectric function ε­(ω) describes how the materials interact with electromagnetic radiation.^46^ The real part, ε_1_(ω), represents polarization effects and energy storage through bound charge displacement, while the imaginary part ε_2_(ω) quantifies energy dissipation and absorption capacity via interband transitions.^47,53^ Figure 11(a–c) illustrates the variation of ε_1_(ω) for CsPbBr_3‑y_X_*y*_ perovskites. Static dielectric constant ε_1_(0) values are listed in Table 3. From Figure 11(a–c), ε_1_(ω) spectra increase gradually with photon energy, peaking in the UV regions and then decreasing at higher energies with oscillatory behavior. The occurrence of negative ε_1_(ω) values in the ultraviolet region indicates metallic-type response, whereas positive ε_1_(ω) corresponds to dielectric behavior.^54^ CsPbBr_2_F displays a prominent ε_1_(ω) peak, suggesting strong electronic polarization response, whereas CsPbF_3_ shows the weakest response within the fluorine series.^35^ In the chlorine series, ε_1_(0) systematically decreases with increasing Cl content. This reduction in the static dielectric response is driven by stronger, more localized Pb–Cl bonding and a more compact crystal lattice, which reduces the low-energy electronic polarizability of the system.^13,15^ The iodinated compounds exhibit higher ε_1_(0) values due to the enhanced polarizability of iodine ions, which promotes dielectric screening and reduces exciton binding energy.^30,55^ All investigated perovskite compositions exhibit this zero-crossover threshold deep in the ultraviolet region, specifically within the 12–15 eV energy range. A clear compositional trend is observed: substituting bromine with more electronegative, smaller anions like chlorine and fluorine shifts the plasma frequency to higher photon energies. This blue shift in plasma frequency is driven by lattice contraction and increased electron localization, which demands higher photon energies to induce collective electronic oscillations. This high-energy plasmonic threshold confirms that these materials retain stable dielectric transparency throughout the visible and near-UV spectrum.

**Figure 11:**
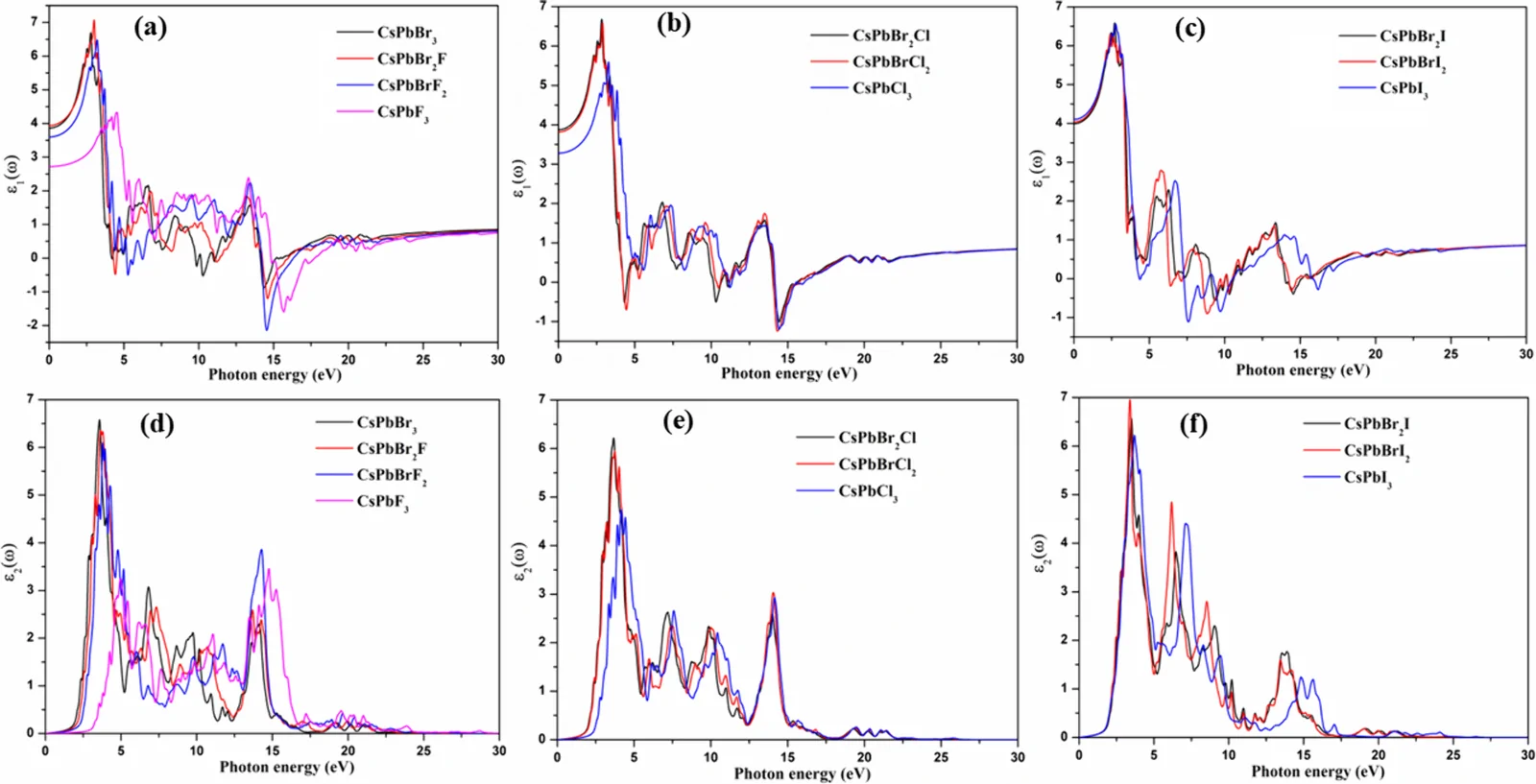
Real ε_1_(ω) *(*a-c) and imaginary ε_2_(ω) (d-f) dielectric spectra of CsPbBr_3‑y_X_*y*_ (X = I, Cl, F; y = 0, 1, 2, 3).

**Table 3:** Calculated Optical Parameters ε_1_(0), Refractive Index *n* (0), and Reflectivity R (0) for CsPbBr_3–*y*_X_*y*_ ^a^

**S. No.**	**System**	**ε**_**1**_ **(0)**	*n* **(0)**	**R (0)**
1	CsPbBr_3_	3.87	1.98	0.12
		4.63 ^53^	1.96 ^15^	0.34 ^30^
		3.91 ^13^	2.15 ^53^	0.13 ^53^
		3.82 ^15^		0.10 ^15^
2	CsPbBr_2_F	3.92	1.99	0.13
3	CsPbBrF_2_	3.69	1.85	0.09
4	CsPbF_3_	2.79	1.65	0.06
		2.71 ^46^	1.64 ^46^	0.08 ^46^
		3.14 ^47^	1.77 ^47^	0.29 ^47^
5	CsPbBr_2_Cl	3.89	1.98	0.12
		3.30 ^13^	1.89 ^15^	0.10 ^15^
		3.57 ^15^		
6	CsPbBrCl_2_	3.85	1.98	0.13
		3.81 ^13^	1.88 ^15^	0.09 ^15^
		3.55 ^15^		
7	CsPbCl_3_	3.32	1.8	0.07
		4.43 ^53^	1.79 ^15^	0.08 ^15^
		3.10 ^13^	2.11 ^53^	0.12 ^53^
		3.23 ^15^		
8	CsPbBr_2_I	4	2	0.12
		5.18 ^13^	2.07 ^30^	0.34 ^30^
		4.29 ^30^		
9	CsPbBrI_2_	4.01	2.01	0.12
		3.92 ^13^	2.06 ^30^	0.3 ^13^
		4.25 ^30^		
10	CsPbI_3_	4.04	2.03	0.13
		5.47 ^13^	2.34 ^30^	0.17 ^53^
		6.00 ^53^	2.45 ^53^	

a X = I, Cl, F; y = 0, 1, 2, 3.

As presented in Table 3, the static dielectric response for the iodine series scales monotonically from 3.87 (CsPbBr_3_) to 4.04 (CsPbI_3_) due to the enhanced polarizability of the larger I ions. Conversely, the chlorine series exhibits a slight nonmonotonic fluctuation at the intermediate CsPbBr_2_Cl composition (3.89) before dropping to 3.32 for pure CsPbCl_3_. This deviation from a purely linear interpolation is driven by the structural phase change from cubic Pm3̅m to tetragonal *P4/mmm*. The resulting directional axial compression (*c/a* < 1) breaks the isotropic dipole framework, inducing localized structural distortions in the Pb-X bonds that slightly modify the electronic polarizability vectors at intermediate mixed concentrations. Figure 11 (d–f) presents ε_2_(ω) for the same set of materials, describing the absorption of incident photons and transition probabilities between valence and conduction bands. The onset of ε_2_(ω) identifies the optical bandgap thresholds. A clear blue shift in these thresholds is observed upon fluorine and chlorine substitution (ranging from 1.98 eV for CsPbBr_2_Cl to 3.25 eV for CsPbF_3_), attributed to lattice contraction and widened bandgaps. Iodinated systems exhibit a red shift due to increased Pb 6*p* – I 5*p* orbital overlap. Beyond these thresholds, ε_2_(ω) intensifies with increasing photon energy and exhibits multiple resonant peaks in the visible region. These peaks correspond to direct interband electronic transitions responsible for light absorption, making these compositions effective for photovoltaic and optoelectronic applications.^46,53^ The optical absorption coefficient α­(ω) was calculated for CsPbBr_3–y_X_*y*_ perovskites, and the corresponding spectra are illustrated in Figure 12 (a–c). The absorption coefficient α­(ω) confirms the semiconducting nature of these materials, with no absorption occurring below the bandgap threshold. All investigated perovskite compositions display strong optical absorption profiles. In the visible light range, the absorption coefficients of the iodine-containing compounds exceed 1 × 10^5^ cm^–1^, confirming their exceptional light-harvesting capability. The primary absorption peaks located in the lower-energy region for the pristine endmembers reach up to 1.15 × 10^6^ cm^–1^ for CsPbI_3_ (at ∼ 7.5 eV), 1.06 × 10^6^ cm^–1^ for CsPbBr_3_ (at ∼ 10.2 eV), 1.01 × 10^6^ cm^–1^ for CsPbCl_3_ (at ∼ 10.8 eV), and 0.72 × 10^6^ cm^–1^ for CsPbF_3_ (at ∼ 11.2 eV). This systematic variation numerically demonstrates the strong optoelectronic influence of direct interband transitions near the band edges as the halide composition changes. Strong absorption features arise when photons promote electrons from valence-band states dominated by halide *p* orbitals to conduction-band states primarily composed of Pb *p* orbitals. Variations in the halide composition modulate the DOS distribution and bandgap width, thus influencing the energy positions and intensities of the absorption peaks. This interplay between the DOS and α­(ω) underlines the impact of halide substitution on the optical properties, further guiding the design of perovskite materials with customized absorption profiles for enhanced photovoltaic performance.

**Figure 12:**
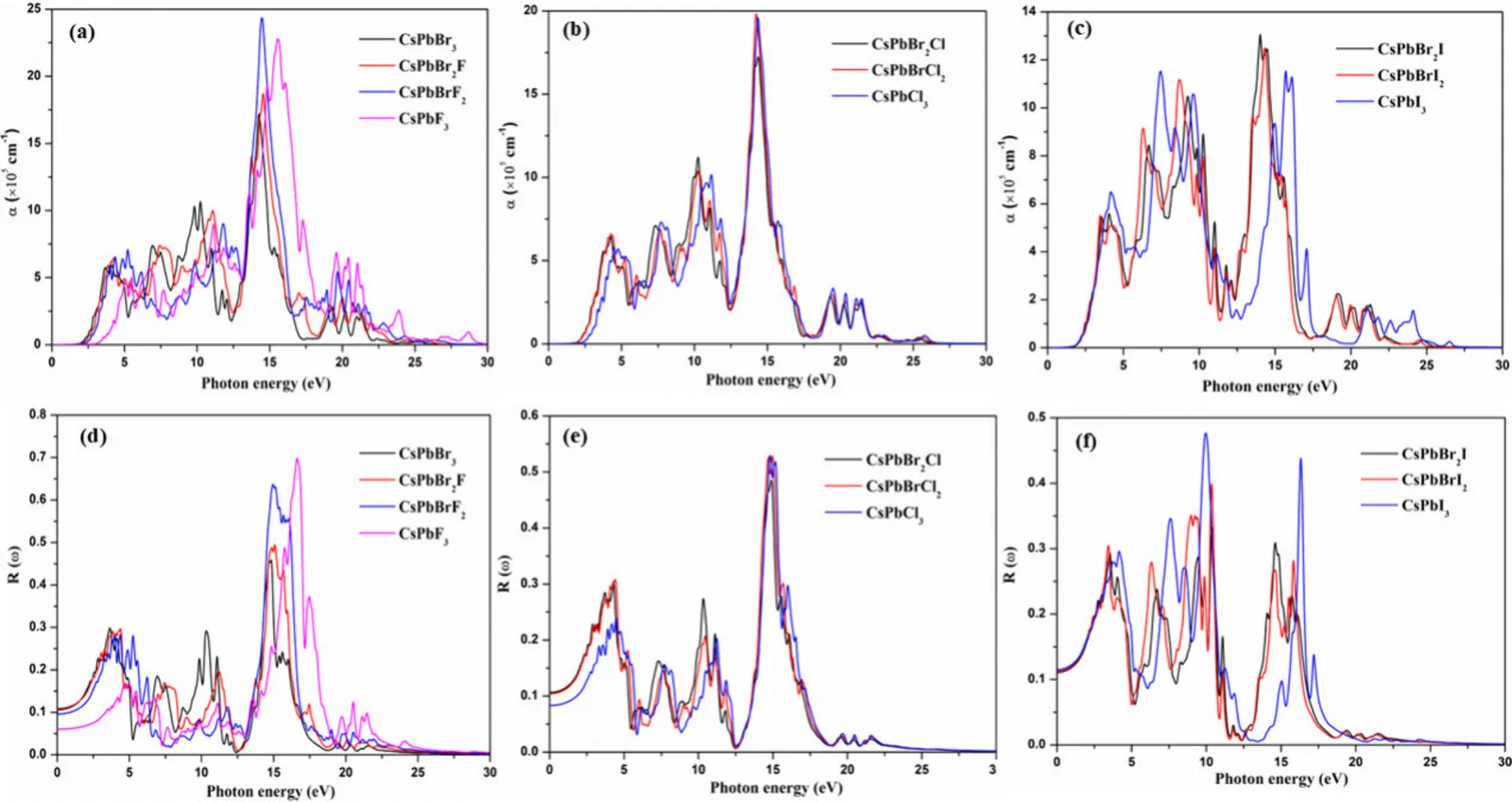
Optical absorption coefficient α­(ω) (a-c) and reflectivity R (ω) (d-f) spectra of CsPbBr_3‑y_X_*y*_ (X = I, Cl, F; y = 0, 1, 2, 3).

The reflectivity R­(ω) spectra as a function of photon energy for CsPbBr_3–y_X_*y*_ perovskites are presented in Figure 12(d-e). Reflectivity quantifies the proportion of incident electromagnetic radiation that is reflected from the material’s surface, providing critical insights into its surface optical behavior. In the low-energy region below the absorption edge, the optical transmittance is defined as T ≈ 1 – R. With a calculated static reflectivity of just 0.06, CsPbF_3_ mathematically yields an excellent theoretical optical transmittance of ∼ 94%. This implies that a mere 6% of incoming photons are lost to front-surface reflections. Conversely, intermediate and iodine-rich variants (such as CsPbI_3_ and CsPbBrI_2_) lose roughly 12 – 13% to reflection, resulting in a lower transmission window. This validates the quantified operational superiority of CsPbF_3_ for transparent protective window layers. Adjustments in halide composition significantly influence reflectivity profiles by altering electronic band structures and surface electronic responses, enabling tunability of optical characteristics to target desired application spectra. The refractive index *n*(ω) is a fundamental optical parameter characterizing how a material modulates the velocity and propagation of incident electromagnetic radiation, influencing transparency and light–matter interactions. For the halide and mixed-halide perovskites studied, the energy-dependent refractive index *n*(ω) shown in Figure 13(a–c) follows the trend of ε_1_(ω), reflecting their intrinsic mathematical relationship. At zero photon energy, the static refractive index values *n* (0) is calculated and listed in Table 3. Following the static point, *n*(ω) increases to a pronounced peak within the infrared and visible regions, indicating strong polarization responses at these energies. Beyond these peaks, the refractive index decreases with increasing photon energy in the ultraviolet (UV) range but remains greater than unity, confirming that photons slow down due to interactions with the electronic environment of the material.^56^ CsPbI_3_ possesses the highest refractive index values, indicating stronger photon-electron coupling, associated with its narrower bandgap and higher polarizability. As summarized in Table 3, the static refractive index *n*(0) values display a minimal shift from 1.98 for CsPbBr_3_ to 2.03 for CsPbI_3_. This narrow variation is physically dictated by the Maxwell relation $n\left(0\right)=\sqrt{\epsilon_{1}\left(0\right)}$ , where the square root function mathematically compresses the underlying increase observed in the static dielectric constants (3.87 for CsPbBr_3_ → 4.04 for CsPbI_3_). Additionally, the enhanced electronic polarizability introduced by the larger I ions is counterbalanced by the systematic expansion of the unit cell volume (214.1 Å^3^ for CsPbBr_3_ → 261.4 Å^3^ for CsPbI_3_), which keeps the net polarizability density per unit volume stabilized across the substitution series.

**Figure 13:**
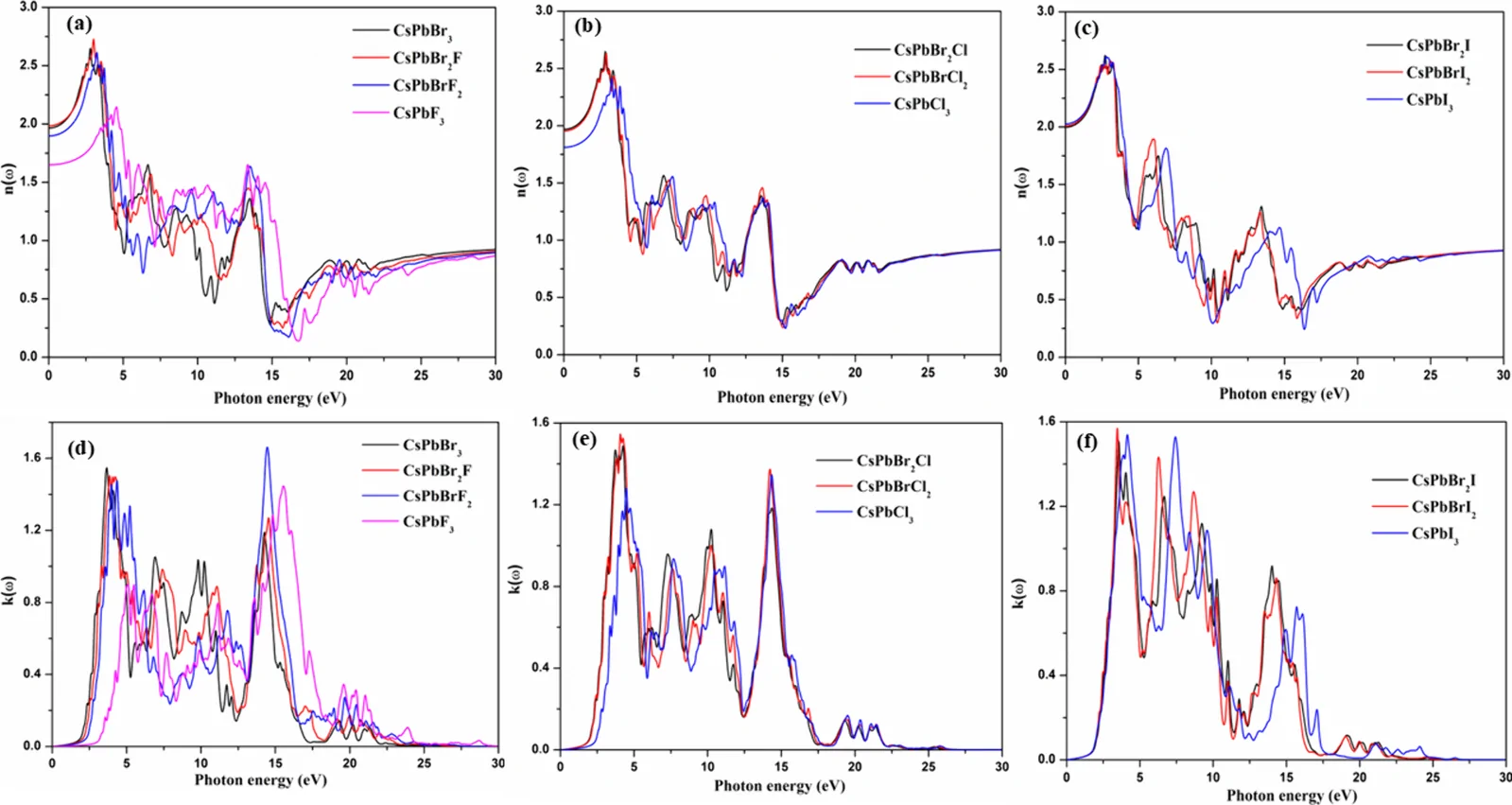
Refractive index *n*(ω) (a-c) and extinction coefficient *k*(ω) (d-f) spectra of CsPbBr_3–*y*_X_*y*_ (X = I, Cl, F; y = 0, 1, 2, 3).

As depicted in Figure 13(d-e), the extinction coefficient *k*(ω) profiles closely mirror the absorption coefficient α­(ω) shown in Figure 12 (a-c), as well as the imaginary part of the dielectric function ε_2_(ω), confirming their intrinsic interdependence. When photon energies surpass the bandgap threshold of these materials, *k*(ω) exhibits a sharp increase, reaching prominent peaks primarily within the visible region of the electromagnetic spectrum. This sharp rise indicates strong light–matter interaction and significant attenuation through electronic transitions from the valence to conduction bands. The highest peaks in *k*(ω) coincide with pronounced absorption features across visible and ultraviolet wavelengths, highlighting the potential of these perovskites for applications in photovoltaics as well as in UV photodetectors.

### 3.5 SLME

The Spectroscopic Limited Maximum Efficiency (SLME) provides a theoretical framework for evaluating the photovoltaic performance of semiconductor absorber materials. Recent work by Patel et al. investigated the thickness-dependent efficiency of CsPbX_3_ absorbers using SLME calculations, demonstrating that optimal thickness varies significantly with halide composition.^57^ Jbara et al. employed DFT to investigate mixed-halide influences on the optoelectronic properties of CsPb­(I/Br)_3_ systems, revealing a gradual bandgap increase with bromide incorporation. These findings align with SLME predictions, which show decreasing maximum efficiencies as bromide content increases.^58^ This study analyzed SLME data for cesium lead halide perovskites and their mixtures. The SLME values were calculated as a function of absorber layer thickness ranging from 0 to 5 μm. Figure 14 represents the calculated SLME values as a function of absorber thickness for the ten cesium lead halide compositions. The data reveals that iodine-rich compositions exhibit the highest SLME; CsPbI_3_ achieves a maximum SLME of approximately 31%, followed by CsPbBrI_2_ (28%) and CsPbBr_2_I (26%). As halide ions progress from fluoride to iodine, the SLME increases monotonically, with CsPbF_3_ showing the lowest value of approximately 5%. The SLME framework natively computes the maximum theoretical conversion efficiency by cross-referencing our calculated optical absorption spectra against the AM1.5G solar reference spectrum. As the electronic bandgaps widen continuously upon F and Cl substitution (3.29 eV for CsPbF_3_ and 2.55 eV for CsPbCl_3_ under mBJ), the absorption edges blue-shift into the high-energy ultraviolet regime. The detailed math yields low efficiencies because the low-energy visible photons, which constitute the bulk of the solar spectrum, contain insufficient energy to excite carriers across these wide gaps, meaning they pass through the material completely unabsorbed. All compositions exhibit characteristic saturation curves where SLME plateaus at an absorber thickness between 2–3 μm. In the context of practical thin-film solar cell manufacturing, a thickness of 2–3 μm represents an upper ceiling for maximum light capture, ensuring complete optical absorption near the band edge. In real-world devices, the operational thickness is typically optimized to be much thinner (typically around 300–500 nm) to balance light absorption with efficient carrier collection. Because metal halide perovskites often face experimental constraints related to short carrier diffusion lengths and nonradiative recombination losses, operating at the theoretical 2–3 μm maximum would lead to severe charge transport resistance.

**Figure 14:**
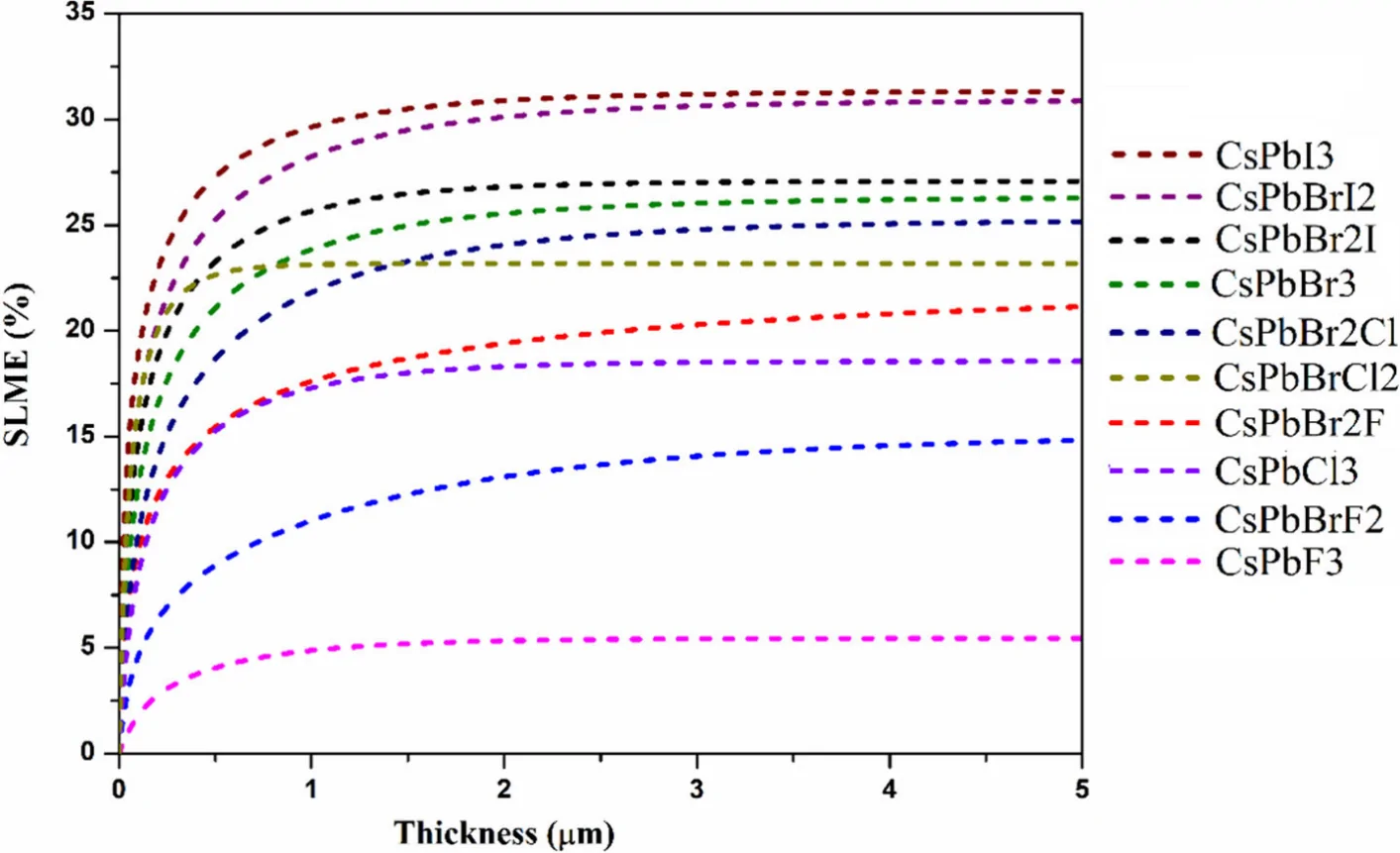
Spectroscopic limited maximum efficiency (η) as a function of absorber layer thickness for various cesium lead halide perovskite compositions.

Therefore, while the 2–3 μm plateau defines the absolute limit for optical thick-film optimization, practical thin-film engineering must prioritize thinner absorber geometries to maximize carrier extraction efficiency. Higher-bandgap materials show a more gradual increase in efficiency relative to thickness, correlating with their lower initial absorption coefficients. Table 4 summarizes the extracted parameters for each composition.

**Table 4:** Calculated SLME (η) Parameter for CsPbBr_3–*y*_X_*y*_ ^a^

**System**	CsPbF_3_	CsPbBrF_2_	CsPbBr_2_F	CsPbCl_3_	CsPbBrCl_2_	CsPbBr_2_Cl	CsPbBr_3_	CsPbBr_2_I	CsPbBrI_2_	CsPbI_3_
**η (%)**	5	12	18	15	20	22	24 24.53 ^57^	26	28	31 30.41 ^57^

a X = I, Cl, F; y = 0, 1, 2, 3.

## 4 Conclusion

This study presents a comprehensive DFT investigation into the structural, electronic, and optical properties of mixed-halide CsPbBr__3_–y_X_*y*_ (X = I, Cl, F; y = 0, 1, 2, 3) perovskites. Structural analysis confirms that the overall unit cell volume contracts continuously with smaller F and Cl ions and expands with larger I ions, driving directional tetragonal distortions (*c/a* < 1) in the mixed phases. Electronic structure analysis demonstrates significant bandgap tunability dictated by halide electronegativity. Substitution with F and Cl widens the bandgap primarily because their more electronegative *p* orbitals lower the valence band maximum. Conversely, iodine substitution narrows the bandgap by raising the valence band edge. Among the computational frameworks employed, TB-mBJ gives a reliable estimate within this structural framework. From an application perspective, F and Cl substitutions decrease the static low-energy dielectric constant and broaden the electronic gaps, positioning them as potential candidates for ultrawide bandgap and UV-reflective coating layers. Iodine incorporation shifts the optical absorption edge into the visible light range, with CsPbI_3_ achieving a maximum theoretical efficiency of approximately 31%. However, it is important to note that the SLME model operates under radiative limits and does not account for extrinsic thin-film constraints such as nonradiative recombination, carrier diffusion bottlenecks, or series resistance. Overall, these insights provide a clear material roadmap for engineering targeted cesium lead mixed-halide perovskite architectures for next-generation photovoltaics and UV optoelectronic technologies.

## Supplementary Material



## Notes

Goldschmidt Tolerance Factor (t_G_), Octahedral Factor (μ), Energy vs Volume (E-V) curve, ENCUT and K-Point Convergence, Calculation of the bandgap bowing parameter and Effective mass calculation (PDF)

## References

[1] Kojima A., Teshima K., Shirai Y., Miyasaka T. (2009). Organometal Halide
Perovskites as Visible-Light Sensitizers for Photovoltaic Cells. J. Am. Chem. Soc..

[2] Lin K., Xing J., Quan L. N., de Arquer F. P. G., Gong X., Lu J., Xie L., Zhao W., Zhang D., Yan C., Li W., Liu X., Lu Y., Kirman J., Sargent E. H., Xiong Q., Wei Z. (2018). Perovskite
Light-Emitting Diodes with External Quantum Efficiency Exceeding 20
per Cent. Nature.

[3] Song J., Li J., Li X., Xu L., Dong Y., Zeng H. (2015). Quantum Dot
Light-Emitting Diodes Based on Inorganic Perovskite Cesium Lead Halides
(CsPbX_3_). Adv. Mater..

[4] Gu L., Tavakoli M. M., Zhang D., Zhang Q., Waleed A., Xiao Y., Tsui K. H., Lin Y., Liao L., Wang J., Fan Z. (2016). 3D Arrays of 1024-Pixel Image Sensors
Based on Lead Halide Perovskite Nanowires. Adv.
Mater..

[5] Ahmadi M., Wu T., Hu B. (2017). A Review on Organic–Inorganic Halide Perovskite
Photodetectors: Device Engineering and Fundamental Physics. Adv. Mater..

[6] Gai C., He D., Wang Y., Wang J., Li J. (2021). Engineering of Halide
Cation in All-Inorganic Perovskite with Full-Color Luminescence. Coatings.

[7] Song Y., Park J. S. (2022). Hybrid Density Functional Theory
Calculation of Orthorhombic
CsPbI_3–3x_Br_3x_ and CsPbBr_3–3x_Cl_3x_. Curr. Appl. Phys..

[8] Ravi V. K., Markad G. B., Nag A. (2016). Band Edge
Energies and Excitonic
Transition Probabilities of Colloidal CsPbX_3_ (X = Cl, Br,
I) Perovskite Nanocrystals. ACS Energy Lett..

[9] Jeon N. J., Noh J. H., Yang W. S., Kim Y. C., Ryu S., Seo J., Seok S. Il. (2015). Compositional Engineering of Perovskite Materials for
High-Performance Solar Cells. Nature.

[10] Stranks S. D. (2017). Nonradiative
Losses in Metal Halide Perovskites. ACS Energy
Lett..

[11] Bati A. S. R., Zhong Y. L., Burn P. L., Nazeeruddin M. K., Shaw P. E., Batmunkh M. (2023). Next-Generation Applications for
Integrated Perovskite Solar Cells. Commun. Mater..

[12] Selmani Y., Bahmad L. (2025). Computational Study of Mixed Halide Perovskites CsSnX_3_ (X = Br, Cl, Mixed Halides) for Optoelectronic and Thermoelectric
Applications: A DFT and Boltzmann Transport Methods. Materials Science and Engineering: B.

[13] Kumari A., Nag A., Kumar J. (2022). Ab-Initio
Study of Halogen Inter-Substituted Perovskite
Cesium Lead Halides for Photovoltaic Applications. J. Phys. Chem. Solids.

[14] Tung J. C., Hsieh Y. H., Liu P. L. (2021). Strain Induced Topological
Insulator
Phase in CsPbBr_x_I_3–x_ (X = 0, 1, 2, and
3) Perovskite: A Theoretical Study. Applied
Sciences (Switzerland).

[15] Ghaithan H. M., Alahmed Z. A., Qaid S. M. H., Aldwayyan A. S. (2020). Structural,
Electronic, and Optical Properties of CsPb­(Br_1–x_Cl_x_)_3_ Perovskite: First-Principles Study with
PBE–GGA and mBJ–GGA Methods. Materials.

[16] Kresse G., Joubert D. (1999). From Ultrasoft Pseudopotentials
to the Projector Augmented-Wave
Method. Phys. Rev. B.

[17] Blöchl P. E. (1994). Projector
Augmented-Wave Method. Phys. Rev. B.

[18] Perdew J. P., Burke K., Ernzerhof M. (1996). Generalized Gradient Approximation
Made Simple. Phys. Rev. Lett..

[19] Tran F., Blaha P. (2009). Accurate Band Gaps for Wide-Gap Semiconductors and Insulators with
a Semilocal Exchange-Correlation Potential. Phys. Rev. Lett..

[20] Tran F., Blaha P. (2017). Importance of the Kinetic
Energy Density for Band Gap Calculations
in Solids with Density Functional Theory. J.
Phys. Chem. A.

[21] Heyd J., Scuseria G. E., Ernzerhof M. (2003). Hybrid Functionals
Based on a Screened
Coulomb Potential. J. Chem. Phys..

[22] Heyd, J. Screened Coulomb Hybrid Density Functionals; PhD Dissertation, Rice University, 2004. 10.1063/1.1668634 15267636

[23] Krukau, A. V. ; Vydrov, O. A. ; Izmaylov, A. F. ; Scuseria, G. E. Influence of the Exchange Screening Parameter on the Performance of Screened Hybrid Functionals. J. Chem. Phys., 2006 125 (22). 10.1063/1.2404663. 17176133

[24] Yang, J. ; Falletta, S. ; Pasquarello, A. Range-Separated Hybrid Functionals for Accurate Prediction of Band Gaps of Extended Systems. NPJ. Comput. Mater. **2023**, 2023 9 (1). 10.1038/s41524-023-01064-x. PMC11621024 39649152

[25] Howard C. J., Stokes H. T. (1998). Group- Theoretical
Analysis of Octahedral Tilting in
Perovskites. Acta Crystallogr..

[26] Stokes H. T., Kisi E. H., Hatch D. M., Howard C. J. (2002). Group-Theoretical
Analysis of Octahedral Tilting in Ferroelectric Perovskites. Acta Crystallographica Section B.

[27] Vajeeston P., Ravindran P., Hauback B. C., Fjellva H. (2011). Prediction of Crystal
Structure, Lattice Dynamical, and Mechanical Properties of CaB_2_H_2_. Int. J. Hydrogen Energy.

[28] Vajeeston P., Ravindran P., Fjellva H. (2012). Prediction of Structural, Lattice
Dynamical, and Mechanical Properties of CaB_2_. RSC Adv..

[29] Ghaithan H. M., Alahmed Z. A., Qaid S. M. H., Aldwayyan A. S. (2020). First Principle-Based
Calculations of the Optoelectronic Features of 2 × 2 x 2 CsPb­(I_1‑X_Br_x_)_3_ Perovskite. Superlattices Microstruct..

[30] Rajeswarapalanichamy R., Amudhavalli A., Padmavathy R., Iyakutti K. (2020). Band Gap Engineering
in Halide Cubic Perovskites CsPbBr_3–y_I_y_ (y = 0, 1, 2, 3) – A DFT Study. Materials
Science and Engineering: B.

[31] Yuan G., Qin S., Wu X., Ding H., Lu A. (2018). Pressure-Induced Phase
Transformation of CsPbI3 by X-Ray Diffraction and Raman Spectroscopy. Phase Transitions.

[32] Donald K. J. (2006). Electronic
Compressibility and Polarizability: Origins of a Correlation. J. Phys. Chem. A.

[33] Islam M. R., Moghal B. K., Moshwan R. (2022). Tuning the
Electronic, Optical, and
Thermal Properties of Cubic Perovskites CsPbCl_3‑n_Br_n_ (n = 0, 1, 2, and 3) through Altering the Halide Ratio. Phys. Scr..

[34] Mahalaxmi P., Balakrishnan K., Veerapandy V., Vajeeston N., Vajeeston P. (2025). First-Principles Investigation of
Pressure-Induced
Structural Phase Transition and Properties of CsPbF_3_ Polymorphs. ACS Omega.

[35] Ijaz
Khan M., Tanveer M., Sana Ullah Sahar M., Gillani S.S.A., Junaid
Zaidi S.M. (2024). A Comprehensive DFT Study on the Structural, Electronic,
Elastic, and Optical Behaviour of CsPbF_3_ under the Effect
of Stress. Results in Optics.

[36] Yan X., Zhang K., Guo C., Lu Y., Du K., Peng C., Hu X., Jia Y., Xu B., Wang R., Duan W., Han H., Song Z., Liu S., Yang F. (2023). Growth and Characterization of All-Inorganic Halide
Perovskite CsPbF_3_ Single Crystals. Crystals (Basel)..

[37] Moreira R. L., Dias A. (2007). Comment on `̀rediction of Lattice Constant in Cubic Perovskites’’. J. Phys. Chem. Solids.

[38] Thomas N. W. (1996). The Compositional
Dependence of Octahedral Tilting in Orthorhombic and Tetragonal Perovskites. Acta Crystallogr..

[39] Woodward P. M. (1997). Octahedral
Tilting in Perovskites. I. Geometrical Considerations. Acta Crystallogr..

[40] Woodward P. M. (1997). Tilting
Octahedral in Perovskites. II. Structure Stabilizing Forces. Acta Crystallogr..

[41] Xian Y., Zhang Y., Rahman N. U., Yin H., Long Y., Liu P., Li W., Fan J. (2020). An Emerging
All-Inorganic CsSn_x_Pb_1‑X_Br_3_ (0 ≤ *x* ≤ 1) Perovskite Single Crystal:
Insight on Structural
Phase Transition and Electronic Properties. J. Phys. Chem. C.

[42] Sabino, F. P. ; Zhao, X. G. ; Dalpian, G. M. ; Zunger, A. Impact of Symmetry Breaking and Spin-Orbit Coupling on the Band Gap of Halide Perovskites. Phys. Rev. B **2024**, 2024 110 (3). 10.1103/PhysRevB.110.035160.

[43] Pazoki M., Imani R., Röckert A., Edvinsson T. (2022). Electronic
Structure of 2D Hybrid Perovskites: Rashba Spin-Orbit Coupling and
Impact of Interlayer Spacing. J. Mater. Chem.
A Mater..

[44] Sun P. P., Li Q. S., Yang L. N., Li Z. S. (2016). Theoretical Insights
into a Potential Lead-Free Hybrid Perovskite: Substituting Pb^2+^ with Ge^2+^. Nanoscale.

[45] Butorin S.
M. (2024). Band Gaps
of Hybrid Metal Halide Perovskites: Efficient Estimation. ACS Appl. Energy Mater..

[46] Murtaza G., Ahmad I., Maqbool M., Aliabad H. A. R., Afaq A. (2011). Structural
and Optoelectronic Properties of Cubic CsPbF_3_ for Novel
Applications. Chin. Phys. Lett..

[47] Mohammed Z. Y., Sami S. A., Salih J. M. (2023). First-Principles
Calculation of Structural,
Electronic, And Optical Properties of Cubic Perovskite CsPbF_3_. East European Journal of Physics.

[48] Ahmad M., Rehman G., Ali L., Shafiq M., Iqbal R., Ahmad R., Khan T., Jalali-Asadabadi S., Maqbool M., Ahmad I. (2017). Structural, Electronic and Optical
Properties of CsPbX_3_(X = Cl, Br, I) for Energy Storage
and Hybrid Solar Cell Applications. J. Alloys
Compd..

[49] Padmavathy R., Amudhavalli A., Manikandan M., Rajeswarapalanichamy R., Iyakutti K., Kushwaha A. K. (2019). Electronic and Optical Properties
of CsSnI_3–y_Cl_y_ (y = 0, 1, 2, 3) Perovskites:
A DFT Study. J. Electron. Mater..

[50] Paul T., Chatterjee B. K., Maiti S., Sarkar S., Besra N., Das B. K., Panigrahi K. J., Thakur S., Ghorai U. K., Chattopadhyay K. K. (2018). Tunable Cathodoluminescence over the Entire Visible
Window from All-Inorganic Perovskite CsPbX_3_ 1D Architecture. J. Mater. Chem. C Mater..

[51] Amudhavalli A., Rajeswarapalanichamy R., Padmavathy R., Iyakutti K. (2021). Electronic Structure
and Optical Properties of CsPbF_3‑y_I_y_ (y
= 0, 1, 2) Cubic Perovskites. Acta Phys. Polym.,
A.

[52] Aktary M., Kamruzzaman M., Afrose R. (2022). A Comparative Study
of the Mechanical
Stability, Electronic, Optical and Photocatalytic Properties of CsPbX_3_ (X = Cl, Br, I) by DFT Calculations for Optoelectronic Applications. RSC Adv..

[53] Murtaza G., Ahmad I. (2011). First Principle Study
of the Structural and Optoelectronic Properties
of Cubic Perovskites CsPbM_3_ (M = Cl, Br, I). Physica B Condens. Matter.

[54] Wang L. J., Kuzmich A., Dogariu A. (2000). Gain-Assisted
Superluminal Light
Propagation. Nature.

[55] Fang Y., Dou S., Shang Q., Wang D., Liu J., Kong X. (2022). Theoretical
Insights into Electronic Structure and NRR Catalytic Mechanism Based
on Halide Perovskites CsPbBr_3‑x_I_x_. Comput. Mater. Sci..

[56] Bigelow N. P., Hagen C. R. (2001). Comment on `̀Observation
of Superluminal
Behaviors in Wave Propagation’’. Phys. Rev. Lett..

[57] Patel, M. J. ; Gupta, S. K. ; Gajjar, P. N. Investigation of Thickness Dependent Efficiency of CsPbX_3_ (X = I, Br) Absorber Layer for Perovskite Solar Cells. J. Phys. Chem. Solids **2023**, 176. 10.1016/j.jpcs.2023.111264.

[58] Jbara A. S., Munir J., Haq B. U., Saeed M. A. (2020). Density Functional
Theory Study of Mixed Halide Influence on Structures and Optoelectronic
Attributes of CsPb­(I/Br)_3_. Appl.
Opt..

