# Supplementary material for: First-Principles Investigation into Structural and Optoelectronic Properties of Mixed Halide Perovskites CsPbBr3–y X y (X = I, Cl, F; y = 0, 1, 2, 3) Using GGA, mBJ, mBJ + SOC, and HSE Functionals
Source: ACS Omega. 2026 Sep 8;11(37):56209–23. doi: 10.1021/acsomega.6c06754 (PMC13613909; doi:10.1021/acsomega.6c06754)
Supplement: Supplementary file 1 [file ao6c06754_si_001.pdf]

Supporting information of "First-principles investigation into structural and optoelectronic properties of mixed halide perovskites $CsPbBr_{3-y}X_y$ (X = I, Cl, F; y = 0, 1, 2, 3) using GGA, mBJ, mBJ + SOC and HSE functionals"

Paraman Mahalaxmi[1], Vasu Veerapandy[1], and Ponniah Vajeeston[2]*

[1]Department of Computational Physics, School of Physics, Madurai Kamaraj University, Madurai 625021, India.

[2]Department of Chemistry, Center for Materials Science and Nanotechnology, University of Oslo, Oslo 0371, Norway.

1. Goldschmidt Tolerance Factor (t_G)

The tolerance factor is a geometric guide used to predict the stability and distortion of the ABX_3 perovskite structure, where:

A-site (Cs^+) is a large cation.

B-site (Pb^{2+}) is a smaller cation.

X-site (F^-, Cl^-, Br^- and I^-) is an anion.

It measures the strain induced by the A-site cation fitting into the BX_6 framework cavity.
It is calculated using the ionic radii of the component ions (r_{Cs}, r_{Pb} and r_X) as follows:

$$t_G = \frac{r_{Cs} + r_X}{\sqrt{2}(r_{Pb} + r_X)} \quad \text{--- (S1)}$$

Optimal Range: $0.8 \leq t_G \leq 1.024$

Predicts the formation of a stable perovskite structure (either cubic, or slightly distorted tetragonal/orthorhombic). The structural stability is maximized when $t \approx 1$.

Outside Range: $t_G < 0.8$ or $t_G > 1.024$

Predicts that the perovskite structure is unlikely to form, or will be highly distorted, leading to the formation of other structural phases[12].

To calculate these values, we will use the following standard Shannon ionic radii for 6-fold coordination (except Cs^+, which is approximated for 12-fold coordination) as is standard practice for halide perovskites (radii in Angstroms, Å)[3]:

Ion	Radius (r)
r_{Cs+} (r_A)	1.81 Å
r_{Pb2+} (r_B)	1.19 Å
r_{F-} (r_X)	1.33 Å
r_{Cl-} (r_X)	1.81 Å
r_{Br-} (r_X)	1.96 Å
r_{I-} (r_X)	2.20 Å

2. **Octahedral Factor (μ)**

The octahedral factor is the ratio of the B-site cation radius (r_B) to the X-site anion radius (r_X):

$$\mu = \frac{r_{Pb}}{r_X} \quad \text{--- (S2)}$$

It specifically determines if the smaller B-site cation (e.g., Pb^{2+}) is the right size to comfortably fit within the cage of six surrounding X^-site anions (e.g., F^-) to form a stable, corner-sharing octahedron.

An ideal value for perfect octahedral coordination is $\mu \approx 0.414$. If μ is outside the stable range (typically $0.44 \leq \mu \leq 0.9$), the BX_6 octahedra are highly strained or unstable, often preventing the formation of the perovskite phase altogether[4].

Table S1. Perovskite Stability Factors of $CsPbX_3$ and mixed halide Series

Material	Halide Composition	r_X (Å)	μ	t_G
$CsPbF_3$	100% F	1.33	0.895	0.881
$CsPbBrF_2$	$\frac{1}{3}$ Br + $\frac{2}{3}$ F	1.54	0.773	0.868
$CsPbBr_2F$	$\frac{2}{3}$ Br + $\frac{1}{3}$ F	1.75	0.68	0.858
$CsPbCl_3$	100% Cl	1.81	0.657	0.853
$CsPbBrCl_2$	$\frac{1}{3}$ Br + $\frac{2}{3}$ Cl	1.86	0.64	0.852

$CsPbBr_2Cl$	$\frac{2}{3}$ Br + $\frac{1}{3}$ Cl	1.91	0.623	0.849
$CsPbBr_3$	100% Br	1.96	0.607	0.844
$CsPbBr_2I$	$\frac{2}{3}$ Br + $\frac{1}{3}$ I	2.04	0.583	0.844
$CsPbBrI_2$	$\frac{1}{3}$ Br + $\frac{2}{3}$ I	2.12	0.561	0.838
$CsPbI_3$	100% I	2.2	0.541	0.834

All ten compositions satisfy the fundamental structural requirements for 3D perovskite formation. However, the systematic decrease in the tolerance factor from $CsPbF_3$ (0.881) to $CsPbI_3$ (0.834) suggests a progressive increase in octahedral tilting and lattice distortion as the average halide radius increases. $CsPbI_3$ exhibits the lowest t_G value, correlating with its experimental tendency toward phase instability at room temperature.

3. **Energy vs. Volume (E-V) curve**

In DFT simulations, codes calculate the total ground-state energy (E) at various volumes rather than pressure directly. Integrating the pressure equation above ($P = -\frac{\partial E}{\partial V}$) yields the Energy-Volume (E-V) Birch-Murnaghan equation:

$$E\,(V) = E_0 + \frac{9V_0B_0}{16}\left\{\left[\left(\frac{V_0}{V}\right)^{2/3} - 1\right]^3 B_0' + \left[\left(\frac{V_0}{V}\right)^{2/3} - 1\right]^2\left[6 - 4\left(\frac{V_0}{V}\right)^{2/3}\right]^3\right\} \text{------------- (S3)}$$

where:

E is the total energy at the given volume V.
E_0 is the minimum energy at the equilibrium volume V_0.
V is the compressed or expanded volume.
V_0 is the equilibrium volume at zero pressure.
B_0 is the Bulk Modulus at zero pressure.
B_0' is the first derivative of the bulk modulus with respect to pressure.

Energy vs. Volume (E-V) curve for the inorganic mixed halide perovskites, generated from DFT calculation and fitted using the Birch-Murnaghan Equation of State (EOS)[5,6]. The calculated E-V curve for $CsPbBr_{3-y}X_y$ (where X = I, Cl, F; y = 0,1,2,3) are given below:

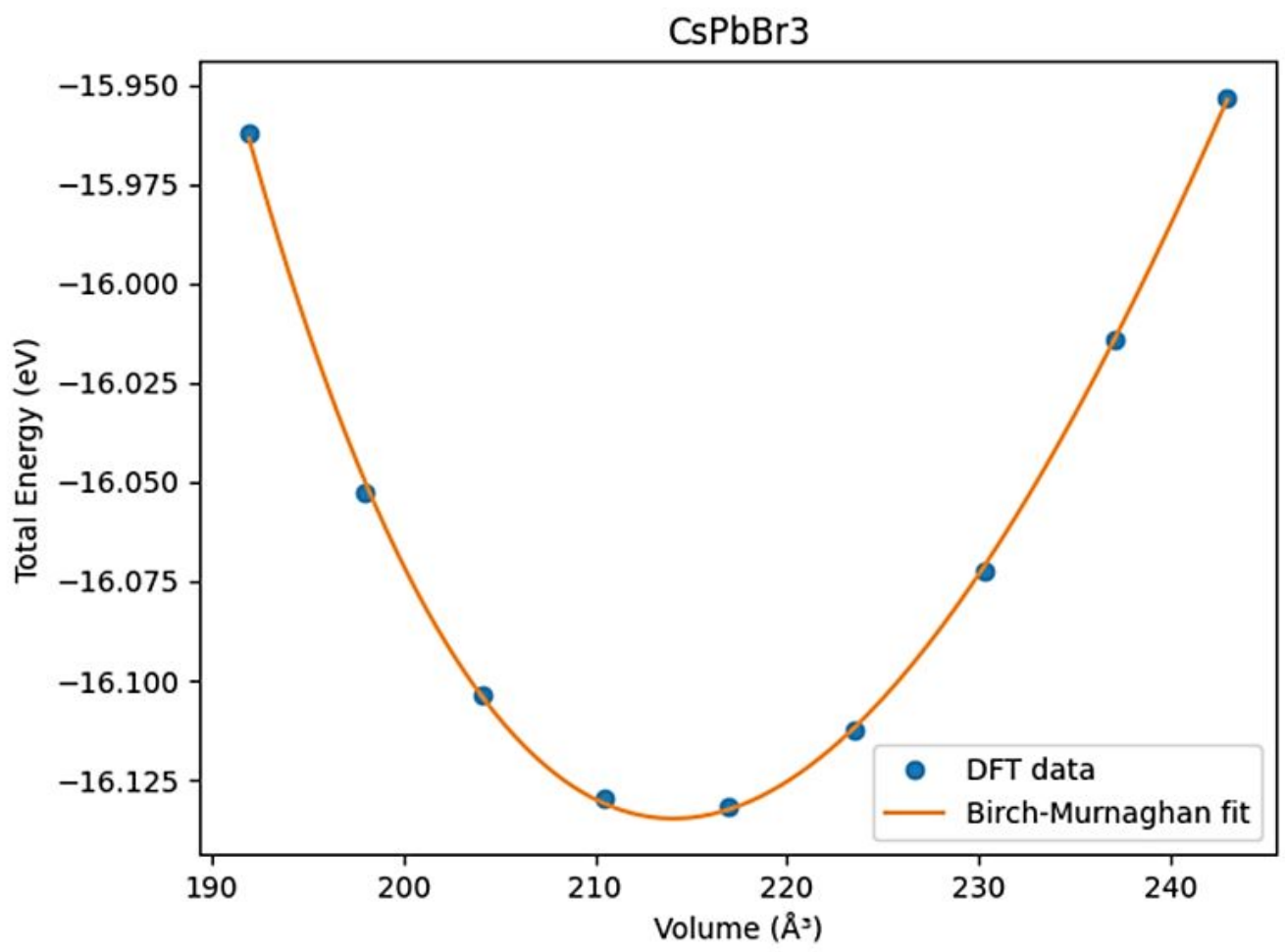

Figure S1. E-V curve of $CsPbBr_3$

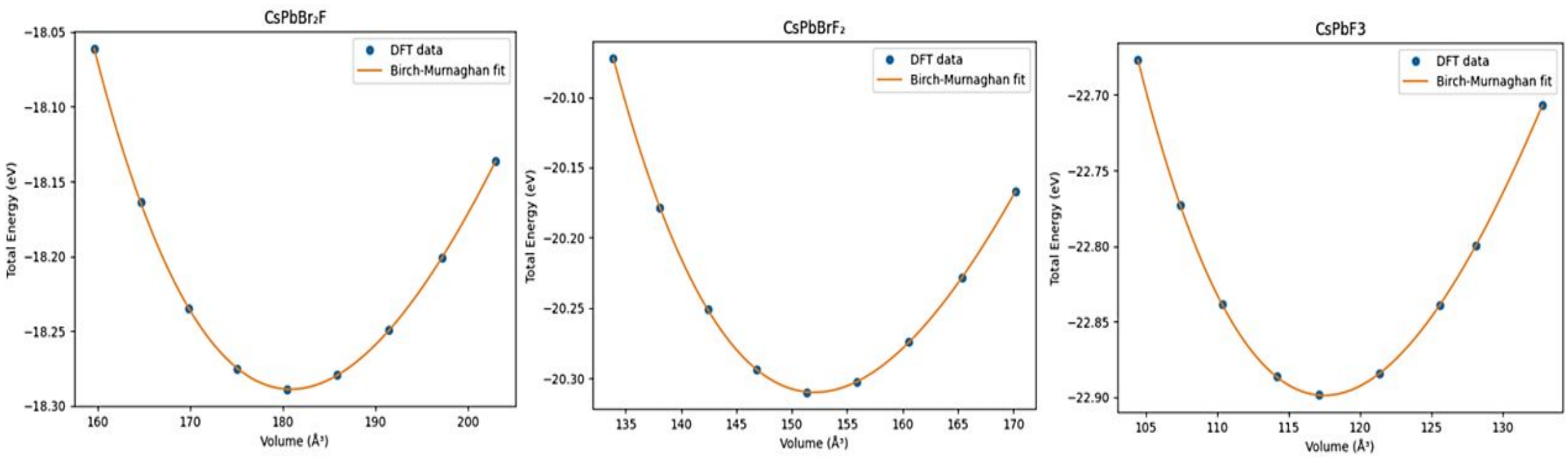

Figure S2. E-V curve of $CsPbBr_2F$, $CsPbBrF_2$ and $CsPbF_3$

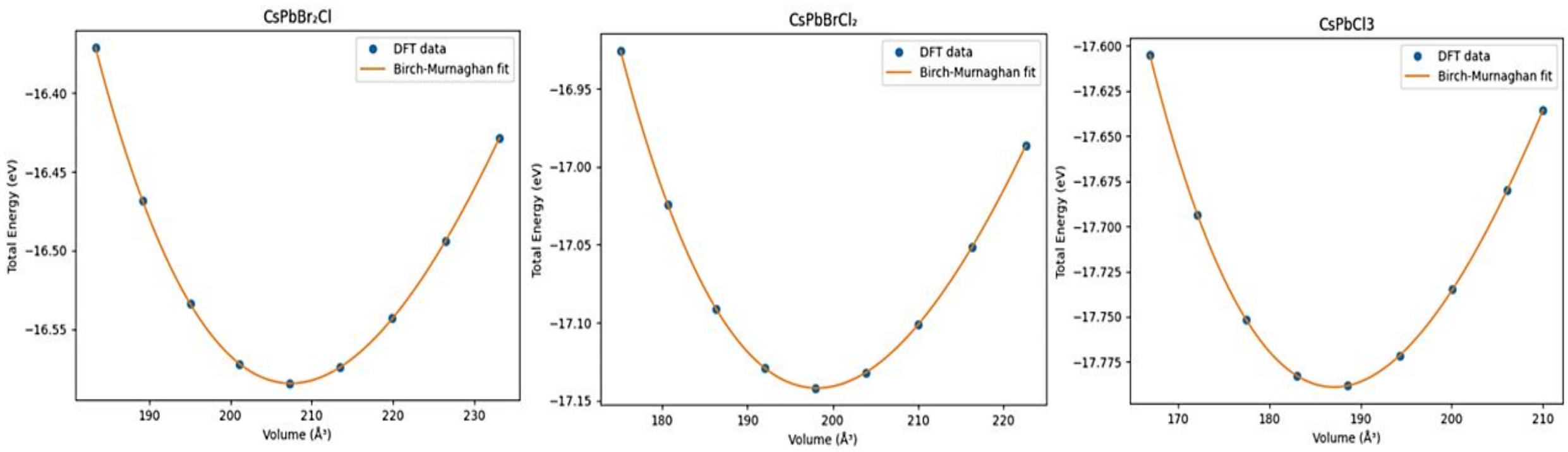

Figure S3. E-V curve of $CsPbBr_2Cl$, $CsPbBrCl_2$ and $CsPbCl_3$

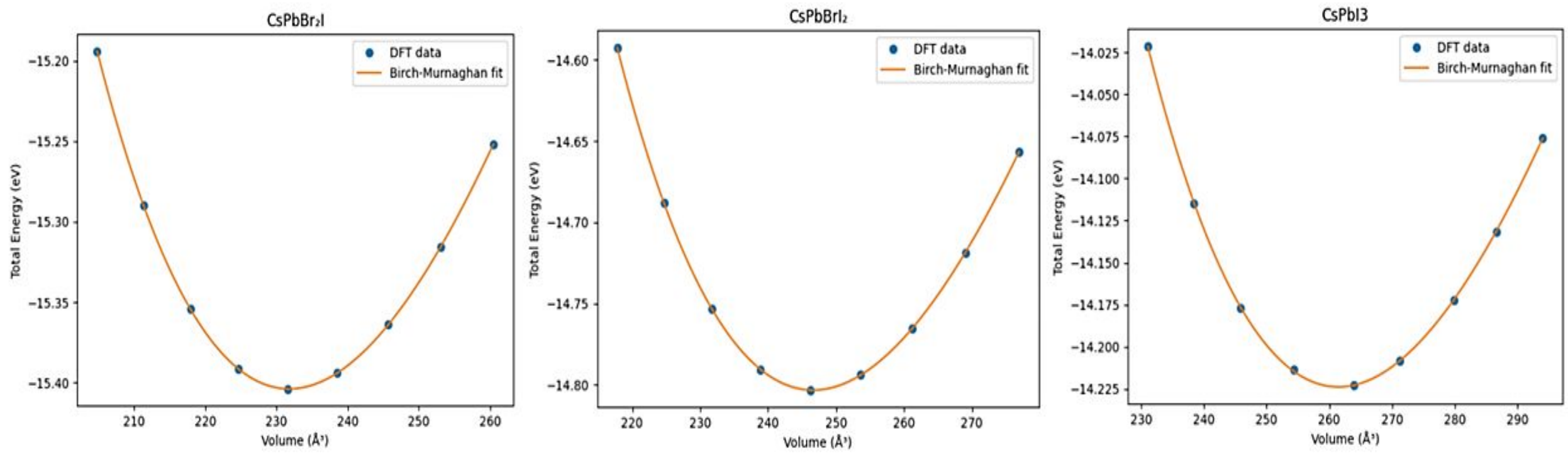

Figure S4. E-V curve of $CsPbBr_2I$, $CsPbBrI_2$ and $CsPbI_3$

4. **ENCUT and K-Point Convergence**

The plane-wave cutoff energy (ENCUT) convergence tests for systems $CsPbBr_3$, $CsPbBr_2F$, $CsPbCl_3$, and $CsPbBrI_2$ are calculated and presented in Figure S5 (a–d). Total energy converges to within 1 meV/atom for ENCUT ≥ 450 eV. Our selected value of 520 eV exceeds the default POTCAR ENMAX by ~30%, ensuring well-converged results.

The k-mesh convergence tests for systems $CsPbBr_3$, $CsPbBr_2F$, $CsPbCl_3$, and $CsPbBrI_2$ are performed with meshes ranging from 2×2×2 to 12×12×12 and presented in Figure S5 (e–h). For structural optimization, a 6×6×6 Monkhorst-Pack k-mesh achieves total energy convergence within 1 meV/atom for all tested systems. The energy difference between 6×6×6 and 8×8×8 is less than 0.5 meV/atom, justifying our choice. For density of states calculations, an 8×8×8 k-mesh was selected as the bandgap converged to within 0.01 eV[7].

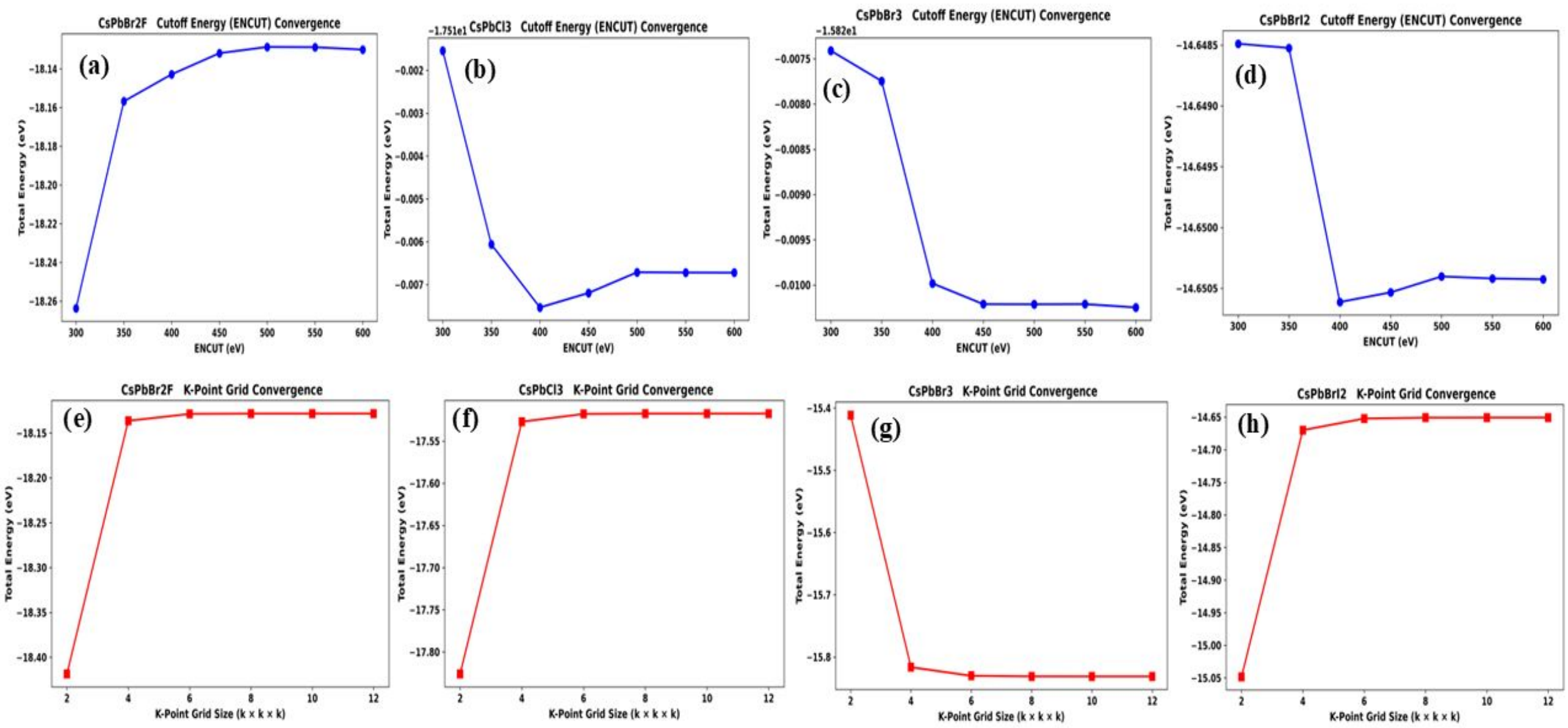

Figure S5. Convergence tests for plane-wave cutoff energy (ENCUT) (a–d) and k-point grid density (e–h) as a function of Total energy for $CsPbBr_2F$, $CsPbCl_3$, $CsPbBr_3$, and $CsPbBrI_2$, respectively.

For optical property calculations, a denser 10×10×10 Monkhorst-Pack k-mesh was employed. Convergence tests on $CsPbBr_3$ confirmed that the 10×10×10 mesh achieves convergence of the static dielectric constant $\varepsilon_1(0)$, and the static refractive index *n* (0) compared to a 12×12×12 reference mesh and given below.

k-mesh	$\varepsilon_1(0)$	*n* (0)
8×8×8	3.84	1.96
10×10×10	3.87	1.98
12×12×12	3.88	1.98

5.**Calculation of the bandgap bowing parameter**

In mixed-halide perovskites, the composition-dependent bandgap *Eg* (*y*) typically deviates from a purely linear interpolation (Vegard's law) and is quantified using the standard quadratic bowing equation:

$$E_g(y) = \left(1 - \frac{y}{3}\right)E_g(\mathrm{CsPbBr_3}) + \frac{y}{3}E_g(\mathrm{CsPbF_3}) - b\left(1 - \frac{y}{3}\right)\frac{y}{3} \text{ ------------------------ (S4)}$$

where *b* is the bandgap bowing parameter[8].

Using the calculated mBJ bandgap values *Eg* ($CsPbBr_3$) = 2.12eV, *Eg* ($CsPbBr_2F$) = 2.33eV, *Eg* ($CsPbBrF_2$) = 2.55eV, and *Eg* ($CsPbF_3$) = 3.29 eV, substituting *y* = 1 into the formula yields an explicit bowing parameter of *b* = +0.81 eV and at *y* = 2 yields *b* = + 1.58. The positive sign of *b* confirms a downward bowing effect, which originates from localized lattice strain, electronegativity differences between Br and F, and non-linear changes in the valence band maximum (VBM) hybridization upon halogen substitution.

6. **Effective mass calculation**

The effective masses of electrons (m_e^*) at the conduction band minimum (CBM) and holes (m_h^*) at the valence band maximum (VBM) were evaluated along the R→Γ direction using parabolic band fitting[9]:

$$m^* = \hbar^2\left(\frac{d^2E(k)}{dk^2}\right)^{-1} \text{ ---------------------------------- (S5)}$$

The effective masses were extracted by fitting the E-*k* dispersion relation using VASPKIT code. As summarized in Table S2, all studied systems display relatively light effective masses

(m_e^*,$m_h^* < 0.65\, m_0$). The I-substituted series demonstrates the smallest effective masses ($m_e^* \approx 0.24\, m_0$ and $m_h^* \approx 0.23\, m_0$ for $CsPbI_3$), pointing to high carrier mobilities and efficient charge extraction. Conversely, F substitution leads to higher effective masses ($0.62\, m_0$ for $CsPbF_3$), reflecting greater localized state densities near the band edges.

Table S2. Calculated effective masses for $CsPbBr_{3-y}X_y$ perovskites along high symmetric path R → Γ in the Brillouin Zone. Values are given in units of the rest electron mass (m_0).

S.No.	System	mBJ bandgap (eV)	Electron (m_e^*)	Hole (m_h^*)
1.	$CsPbBr_3$	2.12	0.30 m_0	0.26 m_0
2.	$CsPbBr_2F$	2.33	0.42 m_0	0.35 m_0
3.	$CsPbBrF_2$	2.55	0.54 m_0	0.50 m_0
4.	$CsPbF_3$	3.29	0.62 m_0	0.44 m_0
5.	$CsPbBr_2Cl$	2.14	0.31 m_0	0.26 m_0
6.	$CsPbBrCl_2$	2.19	0.30 m_0	0.28 m_0
7.	$CsPbCl_3$	2.55	0.38 m_0	0.30 m_0
8.	$CsPbBr_2I$	1.81	0.25 m_0	0.24 m_0
9.	$CsPbBrI_2$	1.79	0.25 m_0	0.23 m_0
10.	$CsPbI_3$	1.75	0.24 m_0	0.23 m_0

Reference

(1) Saparov, B.; Mitzi, D. B. Organic-Inorganic Perovskites: Structural Versatility for Functional Materials Design. *Chem. Rev.* **2016**, *116* (7), 4558–4596. https://doi.org/10.1021/acs.chemrev.5b00715.

(2) Kieslich, G.; Sun, S.; Cheetham, A. K. Solid-State Principles Applied to Organic–Inorganic Perovskites: New Tricks for an Old Dog. *Chem. Sci.* **2014**, *5* (12), 4712–4715. https://doi.org/10.1039/c4sc02211d.

(3) Shannon, R. D. Revised Effective Ionic Radii and Systematic Studies of Interatomic Distances in Halides and Chalcogenides. *Acta Crystallographica Section A* **1976**, *32* (5), 751–767. https://doi.org/10.1107/S0567739476001551.

(4) Li, C.; Lu, X.; Ding, W.; Feng, L.; Gao, Y.; Guo, Z. Formability of ABX_3 (X = F, Cl, Br, I) Halide Perovskites. *Acta Crystallogr. B* **2008**, *64* (6), 702–707. https://doi.org/10.1107/S0108768108032734.

(5) Vajeeston, P.; Ravindran, P.; Hauback, B. C.; Fjellva, H. Prediction of Crystal Structure, Lattice Dynamical, and Mechanical Properties of CaB_2H_2. **2011**, *6*, 1–10. https://doi.org/10.1016/j.ijhydene.2011.05.038.

(6) Karazhanov, S. Z.; Ravindran, P.; Vajeeston, P.; Ulyashin, A.; Finstad, T. G.; Fjellvåg, H. Phase Stability, Electronic Structure, and Optical Properties of Indium Oxide Polytypes. *Phys. Rev. B Condens. Matter Mater. Phys.* **2007**, *76* (7), 1–13. https://doi.org/10.1103/PhysRevB.76.075129.

(7) Ray, D.; Clark, C.; Pham, H. Q.; Borycz, J.; Holmes, R. J.; Aydil, E. S.; Gagliardi, L. Computational Study of Structural and Electronic Properties of Lead-Free $CsMI_3$ Perovskites (M = Ge, Sn, Pb, Mg, Ca, Sr, and Ba). *Journal of Physical Chemistry C* **2018**, *122* (14), 7838–7848. https://doi.org/10.1021/acs.jpcc.8b00226.

(8) Vegard, L.V. Die Konstitution der Mischkristalle und die Raumfullung der Atome. Zeitschrift fur Physik.**1921**.

(9) Ghaithan, H. M.; Alahmed, Z. A.; Qaid, S. M. H.; Hezam, M.; Aldwayyan, A. S. Density Functional Study of Cubic, Tetragonal, and Orthorhombic $CsPbBr_3$ Perovskite. *ACS Omega* **2020**, *5* (13), 7468–7480. https://doi.org/10.1021/acsomega.0c00197.
